# Iron Biofortification of Myanmar Rice

**DOI:** 10.3389/fpls.2013.00158

**Published:** 2013-05-27

**Authors:** May Sann Aung, Hiroshi Masuda, Takanori Kobayashi, Hiromi Nakanishi, Takashi Yamakawa, Naoko K. Nishizawa

**Affiliations:** ^1^Laboratory of Plant Biotechnology, Department of Global Agricultural Sciences, Graduate School of Agricultural and Life Sciences, The University of Tokyo, Bunkyo-ku, Tokyo, Japan; ^2^Laboratory of Plant Cell Technology, Research Institute for Bioresources and Biotechnology, Ishikawa Prefectural University, Nonoichi, Ishikawa, Japan

**Keywords:** iron, anemia, biofortification, nicotianamine, ferritin, OsYSL2, Myanmar rice, rice transformation

## Abstract

Iron (Fe) deficiency elevates human mortality rates, especially in developing countries. In Myanmar, the prevalence of Fe-deficient anemia in children and pregnant women are 75 and 71%, respectively. Myanmar people have one of the highest per capita rice consumption rates globally. Consequently, production of Fe-biofortified rice would likely contribute to solving the Fe-deficiency problem in this human population. To produce Fe-biofortified Myanmar rice by transgenic methods, we first analyzed callus induction and regeneration efficiencies in 15 varieties that are presently popular because of their high-yields or high-qualities. Callus formation and regeneration efficiency in each variety was strongly influenced by types of culture media containing a range of 2,4-dichlorophenoxyacetic acid concentrations. The Paw San Yin variety, which has a high-Fe content in polished seeds, performed well in callus induction and regeneration trials. Thus, we transformed this variety using a gene expression cassette that enhanced Fe transport within rice plants through overexpression of the nicotianamine synthase gene *HvNAS1*, Fe flow to the endosperm through the Fe(II)-nicotianamine transporter gene *OsYSL2*, and Fe accumulation in endosperm by the Fe storage protein gene *SoyferH2*. A line with a transgene insertion was successfully obtained. Enhanced expressions of the introduced genes *OsYSL2*, *HvNAS1*, and *SoyferH2* occurred in immature T_2_ seeds. The transformants accumulated 3.4-fold higher Fe concentrations, and also 1.3-fold higher zinc concentrations in T_2_ polished seeds compared to levels in non-transgenic rice. This Fe-biofortified rice has the potential to reduce Fe-deficiency anemia in millions of Myanmar people without changing food habits and without introducing additional costs.

## Introduction

Iron (Fe) deficiency is one of the most prevalent micronutrient deficiency problems in humans. According to a World Health Organization (WHO) report (WHO, 2002), Fe deficiency affects an estimated two billion people, causing almost one million deaths annually worldwide. Among risk factors for human health, Fe deficiency ranks sixth in developing countries, where it contributes to high rates of mortality (WHO, 2002). Myanmar is one of the countries with critical levels of Fe-deficiency anemia (IDA) (WHO, 2008). The prevalence of IDA is ∼75% in Myanmar children under 5 years of age, ∼26% in adolescent schoolgirls, ∼45% in non-pregnant women, and ∼71% in pregnant women (MOH, 2003). It shows many population groups are strongly impacted by IDA. Young children and women in pregnancy or breast feeding are most frequently and severely affected (WHO, 2002). IDA causes disruptions in brain development in young children and deaths of women in pregnancy and childbirth. It threatens national productivities and lowers the intellectual capacity of human populations (UNICEF and The Micronutrient Initiative, 2004a) in addition to causing coma and death in severe cases of anemia (WHO, 2002). Mineral deficiency lowers the annual productivity of the Myanmar adult work force by an estimated 0.7% of GDP (UNICEF and The Micronutrient Initiative, 2004b).

The main cause of anemia is inadequate dietary Fe intake. Applications exist that address Fe deficiency, including Fe supplementation, Fe fortification, and dietary diversification. Because of financial, cultural, regional, or religious restrictions, these applications are not always successful (Graham et al., 2001; Bouis et al., 2003; Lyons et al., 2003; Timmer, 2003), but a complementary approach to mineral malnutrition termed “biofortification” is proving to be an efficient solution for developing countries (Graham et al., 2001; Bouis, 2003). Biofortification is a process that increases bioavailable concentrations of essential elements in edible portions of crop plants through agronomic intervention or genetic selection (White and Broadley, 2005). Arguments exist to indicate that once mineral-dense lines have been developed, little additional cost will be incurred in incorporating them into ongoing breeding programs. Therefore, biofortification is sustainable and cost-effective, and reaches remote rural populations (Bouis et al., 2003; Timmer, 2003; Welch and Graham, 2005).

Rice is the main staple food crop in Myanmar. The country was the sixth largest rice producer in the world in 2010 (IRRI, 2013). The national rice growing area occupies 8 million ha, representing 63% of the total cultivated landscape (MOAI, 2010); annual rice production is ∼32 million t year^−1^ (MOAI, 2010). Myanmar people are among the highest rice-consumers globally. Average per capita rice consumption is ∼578 g day^−1^ (Kennedy et al., 2002; Maclean et al., 2002), and represents 75% of the caloric intake. Thus, Fe biofortification of Myanmar rice varieties would provide an effective solution to Fe deficiency in the country.

Among available processes that might be used for Fe biofortification, transgenic methodology has potential to be the fastest and most efficient. There were some reports about improved Fe content in rice grain through transgenic procedures. In a first approach, Goto et al. (1999) generated a *japonica* cv. Kitaake transgenic rice with twofold increase in Fe concentration in endosperm (edible part of the rice grain that includes the polished seed) by expressing the soybean *ferritin* gene (*SoyferH1*) in the endosperm under the control of the rice *glutelin* gene (*OsGluB1*) promoter. Furthermore, through this endosperm-specific expression of *ferritin*, twofold increase in Fe in a transgenic *japonica* cv. Taipei 309 (Lucca et al., 2001), 3.7-fold increase in *indica* cv. IR68144 (Vasconcelos et al., 2003) and 2.1-fold increase in *indica* cv. Pusa Sugandhi II (Paul et al., 2012) were reported. In a second approach, overexpression of the nicotianamine synthase gene (*NAS*) increased Fe concentration in polished rice seeds threefold under greenhouse conditions using *japonica* cv. Tsukinohikari (TK) (Masuda et al., 2009a), *japonica* cv. Dongjin (Lee et al., 2009), and *japonica* cv. Nipponbare (Johnson et al., 2011). In a third approach, Fe concentration in polished rice seeds of TK was increased up to threefold through enhancement of Fe(II)-nicotianamine transporter gene (*OsYSL2*) expression under the control of the rice sucrose transporter gene (*OsSUT1*) promoter, leading to elevated expression in the panicle and immature seeds during seed maturation (Ishimaru et al., 2010). Moreover, by combining these three approaches, Masuda et al. (2012) generated “Fer-NAS-YSL2” rice, in which the Fe content was elevated sixfold in greenhouse-grown T_2_ polished seeds and fourfold in paddy field-grown T_3_ polished seeds of TK. Accordingly, we applied this combined approach to the currently cultivated and consumed Myanmar rice variety.

Hiei et al. (1994) reported the first efficient *Agrobacterium*-mediated transformation of *japonica* rice. Using this published procedure, an *Agrobacterium*-mediated transformation methodology with minor modifications has been developed for many rice varieties other than *japonica*, *indica*, and tropical *japonica*, including *indica* cv. Kasalath (model *indica* rice; Saika and Toki, 2010), *indica* cv. RD6 (Thai commercial rice cultivar; Pipatpanukul et al., 2004), *indica* cv. IR64 (IRRI, a well-known high-yield rice cultivar; Rajesh et al., 2008), and *indica* cv. MR219 (Malaysian rice; Sivakumar et al., 2010). Many studies have also demonstrated that transformation efficiencies depend on culture responses of individual varieties (Pipatpanukul et al., 2004; Nishimura et al., 2007; Summart et al., 2008). Therefore, optimization of tissue culture conditions is essential for varieties that regenerate with difficulty (Hiei et al., 1994, 1997). Relevant reports have been published on callus induction and regeneration in *indica* cv. BR-8 (Bangladesh *indica* rice; Amin et al., 2004) and *indica* cv. KDML 105 (Thai aromatic rice; Summart et al., 2008). Nevertheless, no current reports have described transformations of Myanmar rice varieties, and thus no relevant information exists to aid in the selection of Myanmar varieties that would be suitable for this procedure. Similarly, no information exists to guide selection of callus induction and regeneration media that are efficient for regeneration. Accordingly, production of high-Fe Myanmar rice through Fe-biofortification methodology requires analyses of callus induction and regeneration ability in each variety before performing actual rice transformation.

In the present study, we first tested callus induction and regeneration efficiency in 15 popular Myanmar rice varieties. The Paw San Yin variety produced calli readily and had moderate regeneration efficiency when we used the modified methods of Hiei et al. (1994). The main purpose of our research is to produce Fe biofortified Myanmar rice using the currently cultivated and consumed rice variety and come closer to practical application for the needs. Therefore, we used Paw San Yin variety, which is a famous high-quality variety, and presently cultivated and consumed rice variety in Myanmar. We produced high-Fe Myanmar rice through introduction of the Fer-NAS-YSL2 vector, which includes multiple genes for enhancing Fe transportation and accumulation in rice plants (Masuda et al., 2012). The procedures produced elevated gene expression in T_2_ immature seeds, and the Fe concentration in T_2_ polished seeds increased 3.4-fold in comparison with the non-transgenic line.

## Materials and Methods

### Plant materials

We used seeds of 15 Myanmar high-yield or high-quality rice cultivars, which were provided by the Myanmar Rice Research Center (MRRC), Hmawbi, Myanmar, for the regeneration trials. These were Kyaw Zay Ya (V1), Ayar Min (V2), Paw San Yin (V3), Shwe War Htun (V4), Sin Thwe Lat (V5), Yezin Lone Thwe (V6), Thu Kha Yin (V7), Thee Htet Yin (V8), Sin Nwe Yin (V9), Yadana Toe (V10), Hmawbi 2 (V11), Hmawbi 3 (V12), Hmawbi 4 (V13), Hmawbi 5 (V14), and Hmawbi Kauk Nyin Hmwe (V15) (Table 1). *Japonica* rice cv. TK, a well-known variety for efficient rice transformation, was used as the control for callus induction and regeneration trails.

**Table 1:** **Myanmar rice varieties used in callus induction and regeneration trials**.

Variety number	Variety name	Variety type
V1	Kyaw Zay Ya	HYV
V2	Ayar Min	HYV
V3	Paw San Yin	HQV
V4	Shwe War Htun	HYV
V5	Sin Thwe Lat	HYV
V6	Yezin Lone Thwe	HQV
V7	Thu Kha Yin	HYV
V8	Thee Htet Yin	HYV
V9	Sin Nwe Yin	HYV
V10	Yadana Toe	HYV
V11	Hmawbi 2	HQV
V12	Hmawbi 3	HYV
V13	Hmawbi 4	HQV
V14	Hmawbi 5	HYV
V15	Hmawbi Kauk Nyin Hmwe	HYV

HYV, high-yield variety; HQV, high-quality variety. Seeds were provided by the Myanmar Rice Research Center (MRRC) and source of data were from the Ministry of Agriculture and Irrigation (MOAI), Myanmar.

We used Myanmar rice *tropical Japonica* cv. Paw San Yin (V3) (Table 1; Figure S1 in Supplementary Material) for rice transformation and as the non-transgenic control. Seeds from MRRC were sown in a greenhouse and grown to maturity to provide fresh seeds for transgenic procedures.

### Sterilization of rice seeds

Husks were removed from the seeds manually. The brown seeds were first surface-sterilized in 70% ethanol for 5 min and then rinsed thoroughly with distilled water (DW). Then, the seeds were washed in a 2.5% solution of sodium hypochlorite (NaClO; The Clorox Co., Oakland, CA, USA) containing 0.2% Tween 20 (Sigma-Aldrich, St. Louis, MO, USA) as a wetting agent for 30 min in a rotator. After surface sterilization, seeds were rinsed five times with sterile water and used for induction of calli.

### Media used for callus induction and regeneration test

We used N6D, N6D4, N6D6, 2N6, MSre, and MS media for callus induction and regeneration trials. Components of 2N6 and MS media were from Hiei et al. (1994). Components of N6D media were from Ishizaki and Kumashiro (2008). MSre (MS regeneration) media was modified from Toki (1997), and 2 mg l^−1^ of α-naphthaleneacetic acid and 1 mg l^−1^ of kinetin were applied as hormones for regeneration of plants in all regeneration trials and rice transformation. Components of N6D4 and N6D6 medium matched those in N6D except that 2,4-dichlorophenoxyacetic acid (2,4-D) concentrations were 4 and 6 mg l^−1^ instead of 2 mg l^−1^, respectively. Five media combinations tested through the developmental steps from callus induction to plant regeneration were coded as follows: N6D-N6D-MSre-MS, N6D-2N6-MSre-MS, 2N6-2N6-MSre-MS, N6D4-N6D4-MSre-MS, and N6D6-N6D6-MSre-MS. Thus, the code N6D-N6D-MSre-MS indicates the following sequence of media: (i) N6D medium used for callus induction; (ii) induced calli transferred to N6D medium for subsequent callus growth; (iii) calli transferred to MSre medium for regeneration; (iv) final transfer to MS medium for root and shoot growth.

### Callus induction test

After surface sterilization, 15 seeds of each variety were transferred to various media, such as N6D, N6D4, N6D6, or 2N6, for callus induction and kept in darkness in a growth chamber at 28°C. After 28–36 days, about 30–40 calli that were growing well were transferred to new N6D, N6D4, N6D6, or 2N6 medium and cultured for 10–19 days for subsequent callus growth. During both callus induction and growth stages, we observed callus condition daily and photographed the specimens twice a week. We then assessed callus quantity (the amount of callus induction) and quality (callus yellowness, size, and hardness) for each variety on N6D, N6D4, N6D6, and 2N6 media to determine the best medium combination.

### Plant regeneration test

After callus induction, we transferred all calli except those that were brownish onto MSre medium and cultured them for 11–32 days under constant light at 28°C in a growth chamber until green spots appeared; green spots indicated the germination of somatic embryos. Subsequently, all calli were transferred to MS medium and kept for 5–22 days under constant light conditions at 28°C in a growth chamber until plant length exceeded 3–5 cm. We then opened the culture dishes and added water, after which plants were acclimated for about 3 days in a growth chamber at 28°C with illumination. We counted the numbers of regenerated plants and also recorded the duration of time from callus induction to the appearance of green spots. The condition of each callus and regenerated plant was observed daily and photographed twice a week. Once the plant development stage had been reached, we calculated regeneration efficiency for each variety as follows: regeneration efficiency = total number of regenerated plants/number of callus-induced seeds

### Transformation of the Paw San Yin rice variety

We used the Fer-NAS-YSL2 vector (Masuda et al., 2012) for rice transformation (Figure S2 in Supplementary Material). *Agrobacterium tumefaciens* (strain C58) was used to introduce the construct into *Oryza sativa* L. cv. Paw San Yin (V3) following the modified method outlined in Hiei et al. (1994) and Akiyama et al. (1997).

For Paw San Yin-Fer-NAS-YSL2 transformation, calli were induced for 29 days and preincubated for 8 days in N6D medium. We performed *Agrobacterium*-infection on 2N6-AS medium for 3 days following Hiei et al. (1994) with *Agrobacterium* optical density (OD) concentrations of 0.1 or 0.01. Calli were then treated with first, second, and third media selected by the hygromycin check test: N6D-CH10 (N6D medium containing 500 mg l^−1^ claforan; Sanofi K. K., Tokyo, Japan and 10 mg l^−1^ hygromycin B; Wako, Osaka, Japan) medium for 14 days, N6D-CH30 (N6D medium containing 500 mg l^−1^ claforan and 30 mg l^−1^ hygromycin B) medium for 14 days, and N6D-CH50 (N6D medium containing 500 mg l^−1^ claforan and 50 mg l^−1^ hygromycin) medium for 7 days at 28°C in darkness. High-quality calli were selected and transferred to MSre-CH50 (MSre medium containing 500 mg l^−1^ Claforan and 50 mg l^−1^ hygromycin B) medium and incubated for 48 days at 28°C under illumination. High-quality calli were transferred to MS-C medium (MS medium containing 500 mg l^−1^ claforan and no hygromycin B) and maintained until green spots and regenerated plants appeared after 15 days. Four regenerated plants were obtained and one independent Fer-NAS-YSL2 line of T_0_ transgenic specimens with all inserted genes was selected.

### Detection of the gene insertion in transgenic lines

Leaves of T_0_ transgenic and non-transgenic lines were cut off with scissors and crushed in a Multi-beads Shocker (Yasui Kikai, Osaka, Japan). Subsequently, we prepared total DNA following Ikeda et al. (2001). We performed genomic PCR analysis to check for the inserted gene. Rice endogenous *OsActin1* was detected as a positive control using *OsActin1* forward primer (5′-ACA CCG GTG TCA TGG TCG G-3′) and *OsActin1* reverse primer (5′-ACA CGG AGC TCG TTG TAG AA-3′). The *OsGlb* promoter–*SoyferH2* cassette was detected using the *OsGlb1* promoter forward primer (5′-CCG ATC GCC ATC ATC TCA TCA TCA G-3′) and *SoyferH2* reverse primer (5′-GCT TCC ACC AAC ACC CTT AGA AAG-3′). The *OsActin1* promoter–*HvNAS1* cassette was detected using the *OsActin1* promoter forward primer (5′-GGG TAG AAT TTG AAT CCC TCA GCA-3′) and *HvNAS1* reverse primer (5′-CGA TCT TCT CGA TCA GAG CAG CGA-3′). *HPT* was detected using the *HPT* forward primer (5′-CGG CAT CTA CTC TAT TCC TTT GC-3′) and *HPT* reverse primer (5′-GTC TCC GAC CTG ATG CAG CTC-3′). *NPTII* was detected using the *NPTII* forward primer (5′-GAT GGA TTG CAC GCA GGT TCT C-3′) and *NPTII* reverse primer (5′-GCC AAC GCT ATG TCC TGA TAG C-3′). *iGUS* was detected using the *iGUS* forward primer (5′-CTG TGG AAT TGA TCA GCG TTG G-3′) and *iGUS* reverse primer (5′-CGC AAG TCC GCA TCT TCA TGA C-3′).

### Greenhouse cultivation

Paw San Yin T_0_ transgenic and T_1_ plants were cultivated in a mixture of 3 kg/pot of commercially supplied soil used in Japanese rice nurseries (Bonsolichigou; Sumitomo Chemicals, Tokyo, Japan) and 210 g/pot of vermiculite (Buiesu-Kakou Co., Ltd., Tokyo, Japan). The nutrient content of the bonsol soil were 1.8 g/pot of nitrogen (N), 4.8 g/pot of phosphate (P), and 2.6 g/pot of potassium (K). Slow-release fertilizer (LongTotal-70 and -140; JCAM AGRI. Co., Ltd., Tokyo, Japan) was applied at planting time and panicle initiation time. The nutrients included in the slow released fertilizers per one application time were as follows: N: 0.91 g/pot, P: 0.77 g/pot, K: 0.91 g/pot, Mn: 7 mg/pot, boron (B): 4.2 mg/pot, Fe: 14 mg/pot, Cu: 3.5 mg/pot, Zn: 1.05 mg/pot, magnesium (Mg): 140 mg/pot and molybdenum (Mo): 1.4 mg/pot. Plants were grown in a greenhouse at 30°C with 14 h day^−1^ of natural light, and at 25°C with 10 h day^−1^ of natural light. Specimens were grown in plant cultivation pots (size: 1/5000; Fujiwara-Seisakusho, Tokyo, Japan). One plant was transferred to each pot.

### Quantitative real-time RT-PCR analysis

Total RNA was extracted from immature T_2_ seeds (seeds at an early milky stage, 10 days after fertilization) harvested from each subline and NT line grown in a greenhouse. Seeds were crushed in a Multi-beads Shocker^®^. We subsequently extracted RNA using an RNeasy Plant Mini Kit (Qiagen KK, Tokyo, Japan). First-strand cDNA was synthesized using a ReverTra Ace kit (Toyobo, Osaka, Japan) with oligo-d(T)_30_. Real-time RT-PCR was performed using a StepOnePlus^TM^ Real-Time PCR System (Applied Biosystems, Tokyo, Japan) with SYBR Premix Ex Taq II reagent (Takara, Shiga, Japan). The primers used were as follows: *OsYSL2* forward (5′-GAG GGA CAA CGG TGT CAT TGC TGG T-3′) and *OsYSL2* reverse (5′-TGC AGA AAA GCC CTC GAC GCC AAG A-3′) for *OsYSL2* expression, *HvNAS1* forward (5′-GGA CGT CGC CGA CCT CAC CCA G-3′) and *HvNAS1* reverse (5′-CAG GGA CGC CCC CTC CAC C-3′) for *HvNAS1* expression, and *SoyferH2* forward (5′-GCT TTT ATC TCT CGC CCG TTG-3′) and *SoyferH2* reverse (5′-CAT TGT GTG CAA TCG GAA CAG C-3′) for *SoyferH2* expression. Transcript levels were normalized to the expression levels of alpha-*Tubulin* determined using the primers alpha-*Tubulin* forward (5′-TCT TCC ACC CTG AGC AGC TC-3′) and alpha-*Tubulin* reverse (5′-AAC CTT GGA GAC CAG TGC AG-3′). The sizes of the amplified fragments were confirmed by agarose gel electrophoresis.

### Metal concentration analysis of seeds

We harvested T_1_ and T_2_ seeds from the greenhouse and measured metal concentrations in brown seeds, polished seeds, and husks. We converted brown seeds to polished seeds using a Multi-Beads Shocker, as previously described by Masuda et al. (2009a) with modification for *indica* rice polishing. We transferred 20 seeds to a 2-ml plastic tube (Yasui Kikai, Osaka, Japan) and machine-shook them vigorously through four cycles of 2500 rpm for 300 s. We used three replicates of 10 well-polished seeds each for subsequent analyses. Each 10-seed replicate was dried overnight and weighed before digestion. Subsequently, we added 1 ml of 13 M HNO_3_ (Wako) and 1 ml of 8.8 M H_2_O_2_ (Wako) to each liner to digest the seeds inside. We set all the liners in a MARSS XPRESS digester (CEM Japan, Tokyo, Japan) and digested seeds at 220°C for 20 min. After digestion, samples were collected and made up to 5 ml volume with 0.1 mM HCl and filtered as previously described by Masuda et al. (2009a). Subsequently, we measured metal concentrations in digested seed samples using an inductively coupled plasma atomic emission spectrometer (ICPS-8100; Shimadzu, Kyoto, Japan) and calculated seed metal concentrations.

## Results

### Callus induction and growth at standard 2,4-D concentrations

**Figure 1:**
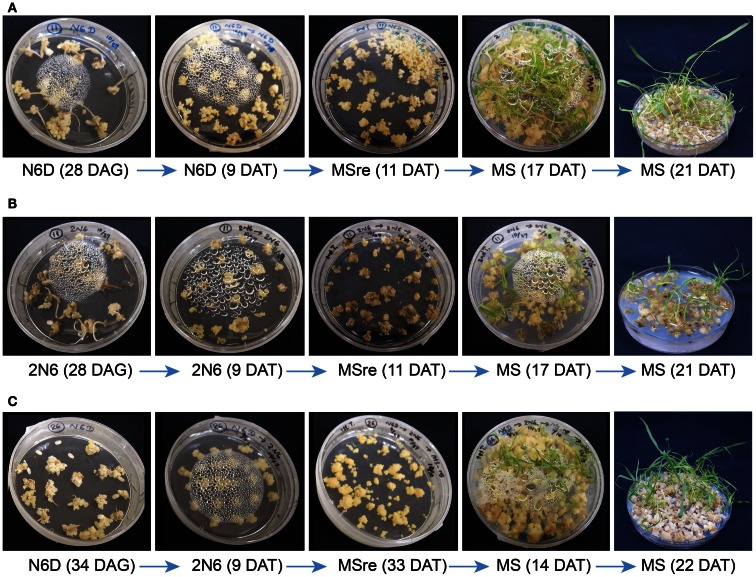
**Callus induction and regeneration efficiency in rice varieties Paw San Yin (V3) and Tsukinohikari in different medium combinations**. **(A)** Tissue culture of Paw San Yin (V3) in the N6D-N6D-MSre-MS medium combination. **(B)** Tissue culture of Paw San Yin (V3) in the 2N6-2N6-MSre-MS medium combination. **(C)** Tissue culture of Tsukinohikari in the N6D-2N6-MSre-MS medium combination. See “Media Used for Callus Induction and Regeneration Test” in the Section “Materials and Methods” for medium combination. DAG, days after germination; DAT, days after transferring. The numerals shown inside parentheses mean DAG or DAT when photograph was taken on each medium.

Fifteen Myanmar rice varieties were included in the callus induction and regeneration trials. Calli were induced on both N6D and 2N6 media. Callus induction capacity differed greatly among the varieties (Figure S3 in Supplementary Material). Within varieties, culture conditions also influenced callus formation. In most varieties, callus induction and callus growth were better on N6D media than on 2N6 media (Figures S3 and S4 in Supplementary Material). Callus induction and formation of Paw San Yin (V3) (especially on N6D-N6D medium) was among the best in Myanmar varieties tested, with performance matching that of the *japonica* cultivar TK (Figure 1, Figures S3 and S4 in Supplementary Material). Callus initiation in the Paw San Yin variety began 8 days after germination (DAG) on all media, and callus proliferation increased after transfer to new medium, especially on N6D (Figures 1A,B).

### Regeneration testing after callus induction at standard 2,4-D concentrations

We observed the regeneration efficiency of 15 Myanmar rice varieties in various medium combinations. For many varieties, including V2, V4, V6, V9, and V13, calli obtained by 2N6-2N6 treatment turned brown and died on MSre media 15–31 days after transferring (DAT), and no regenerated plants were obtained (Figures S5–S9 in Supplementary Material). Plants regenerated only on the N6D-N6D-MSre-MS medium combination. Regenerated plants of varieties V3 and V14 were obtained on both N6D-N6D-MSre-MS and 2N6-2N6-MSre-MS medium combinations (Figure 1, Figure S10 in Supplementary Material). Callus quantity and quality of the Paw San Yin high-quality variety (V3) were similar to those of the TK cultivar in both the callus induction and growth stages, though were somewhat inferior after transfer to MSre regeneration media (Figure 1). However, green spots appeared afterward, and callus proliferation increased considerably after transfer to MS medium. Like several other varieties, many more regenerated plants of V3 developed 21 DAT under the N6D-N6D-MSre-MS medium combination than under the 2N6-2N6-MSre-MS combination (Figure 1).

### Effects of 2,4-D concentrations on callus induction and regeneration on N6D and 2N6 media

Callus inductions of Myanmar rice varieties V1, V8, V10, and V11 were not adequate on either 2N6 or N6D medium (Figures S11A,B–S14A,B in Supplementary Material). 2,4-D concentration is an important callus induction factor in all rice varieties (Bajaj, 1991). Accordingly, we tested the effects of 2,4-D concentrations on callus induction and growth of selected varieties, aiming to improve callus induction and regeneration efficiency. We tested various 2,4-D concentrations, e.g., 4 and 6 mg l^−1^ in addition to the standard concentration of 2 mg l^−1^.

Some elevated 2,4-D concentrations in N6D media enhanced callus induction and growth, and raised the regeneration efficiency in V1 (N6D6-N6D6-MSre-MS), V8 (N6D4-N6D4-MSre-MS), V10 (N6D4-N6D4-MSre-MS), and V11 (N6D6-N6D6-MSre-MS) (Figures S11–S14 in Supplementary Material). V5 and V7 were also tested at 2,4-D concentrations of 4 and 6 mg l^−1^, but callus production was poor and no regenerated plants were produced (data not shown).

### Identification of THE best medium combination and regeneration efficiency

Best culture types in which regenerated plants were obtained for each variety, period kept in each medium from callus induction to acclimation, and numbers of regenerated plants are presented in Table S1 in Supplementary Material. During both callus induction and growth stages, we assessed callus quantity (degree of callus induction) and quality (callus yellowness, size, and hardness). Scores for diverse culture media within and among varieties were used to identify the best culture conditions. Among the 15 Myanmar rice varieties tested, Paw San Yin produced best calli and scored best in these assessments. Relationships between callus condition and the number of regenerated plants in the best medium for each variety are presented in Figure S15 in Supplementary Material.

**Figure 2:**
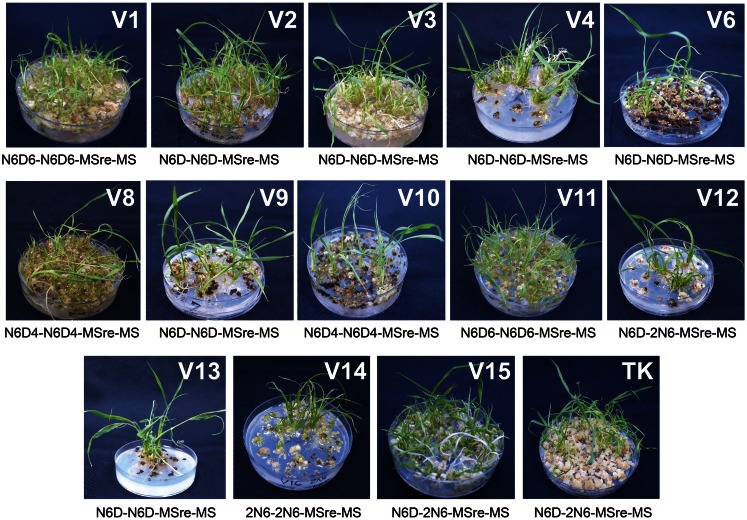
**Identification of the best medium combination for each variety**. V1–V15 shown on upper right part of each picture represent the variety numbers described in Table 1 and TK means Tsukinohikari. The media combination described under each picture showed the best media combination for each variety. Pictures of regenerated plants were taken at 3 days after acclimation. Pictures of V5 and V7 were not shown as regenerated plants were not obtained from these two varieties.

**Figure 3:**
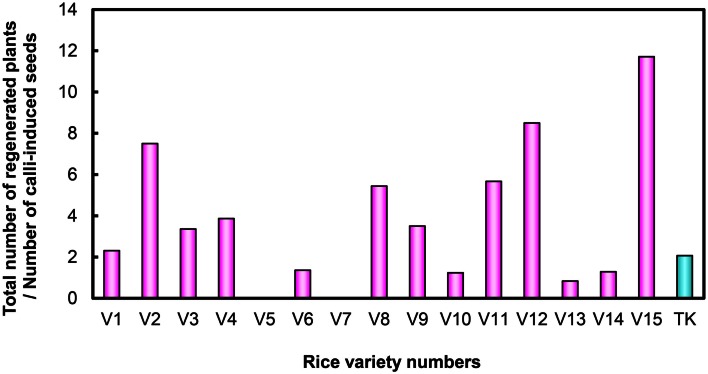
**Regeneration efficiency of Myanmar rice varieties and the Tsukinohikari variety**. V1–V15 under horizontal bar represent the rice variety numbers described in Table 1 and TK means Tsukinohikari. Numbers on vertical bars are quotients of total numbers of regenerated plants divided by numbers of callus-induced seeds, which represent regeneration efficiency.

After examining performance in five types of medium combination, we identified the best medium combination for each variety (Figure 2). Among the 15 Myanmar rice varieties tested, 13 varieties were able to regenerate (Figure 2; Table S1 in Supplementary Material). We calculated regeneration efficiency for each variety in the best medium among those tested (see Plant Regeneration Test in the Materials and Methods; Figure 3). Regeneration efficiency was very different among varieties. Most regenerated well and some exceeded rates of the cultivar TK. Yezin Lone Thwe (V6), Yadana Toe (V10), Hmawbi 4 (V13), and Hmawbi 5 (V14) regenerated poorly; Ayar Min (V2), Hmawbi 3 (V12), and Hmawbi Kauk Nyin Hmwe (V15) regenerated well. Paw San Yin (V3) had intermediate regeneration efficiency that was higher than TK.

**Figure 4:**
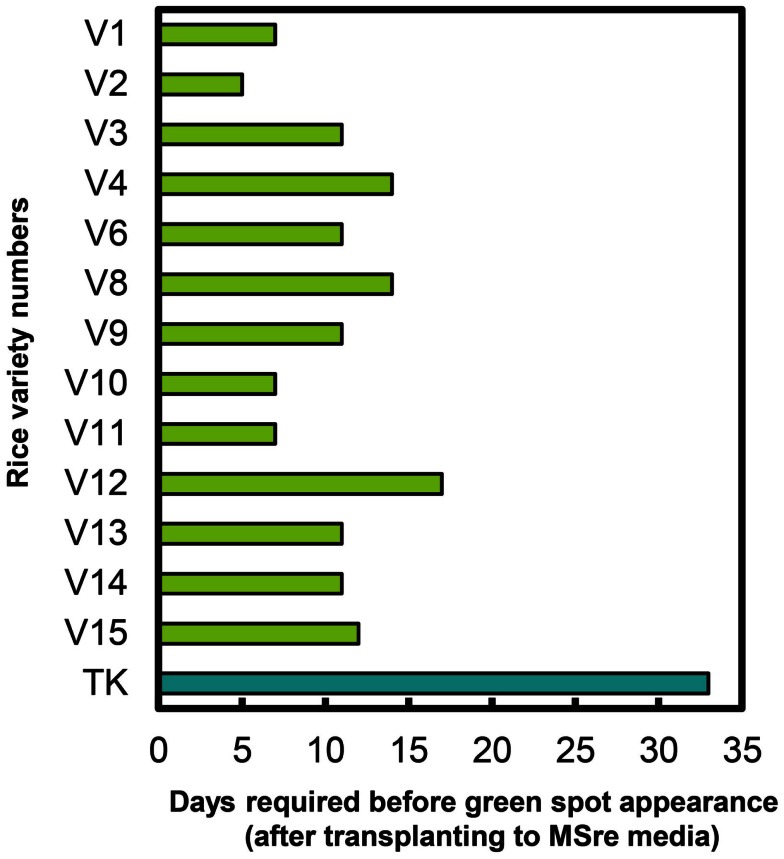
**Durations of time required for green spot appearance in Myanmar rice varieties and in the variety Tsukinohikari**. V1–V15 on vertical bar represent the variety numbers described in Table 1 and TK means Tsukinohikari. Durations of time from transfer to MSre media to green spot appearance are indicated. Regenerated plants were obtained from these green spots.

We also recorded time to green spot appearance on regeneration media (MSre). All 13 regenerating Myanmar rice varieties produced green spots on calli sooner than cultivar TK (Figure 4). Green spots developed most quickly in Ayar Min (V2).

### Transformation of Paw San Yin-Fer-NAS-YSL2

**Figure 5:**
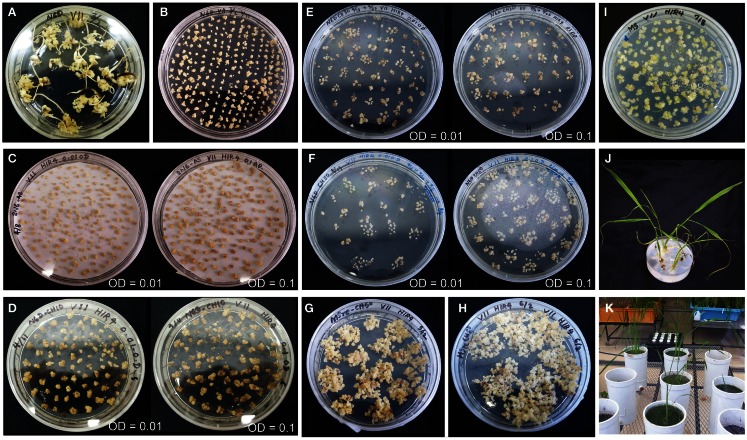
**Transformation of Paw San Yin-Fer-NAS-YSL2**. **(A)** Callus induction (at 29 DAG). **(B)** Pre-incubation (at 8 DAT). **(C)** Three days following *Agrobacterium*-infection. **(D)** Calli on 1st selection medium of N6D-CH10 (at 7 DAT). **(E)** Calli on 2nd selection medium of N6D-CH30 (at 14 DAT). **(F)** Calli on 3^rd^ selection medium of N6D-CH50 (at 7 DAT). **(G)** Calli on MS regeneration medium (MSre-CH50) (at 20 DAT). **(H)** Calli on MSre-CH50 (at 32 DAT). **(I)** Green spots appearance of calli and shoots emergence on MS medium (at 4 DAT). **(J)** Regenerated plants on MS medium. **(K)** Greenhouse-grown T_0_ plants. Left panels of **(C–F)** show transformation with *Agrobacterium* at a concentration of OD = 0.01; right panels of **(C–F)** shows transformation at a concentration of OD = 0.1. The numerals shown with DAG or DAT mean DAG or DAT when photograph was taken on each medium.

We selected the high-quality variety Paw San Yin (V3) for transformation based on its superior callus induction capacity and moderate regeneration efficiency. In addition, Fe content in its polished seeds was also the third highest (2.2 μg g^−1^) among 15 high-yield and high-quality Myanmar varieties tested (data not shown). Callus induction of Paw San Yin on N6D media took 29 days (Figure 5A). Callus condition of Paw San Yin was the best among the varieties tested. Paw San Yin calli were hard, yellow, and larger than those of most other Myanmar rice varieties (Figures 1A and 5A,B, Figures S3 and S4 in Supplementary Material). Paw San Yin calli were smaller than those of TK, but callus induction rates were similar in the two varieties (Figures 1 and 5A). *Agrobacterium*-infection was performed at OD concentrations of 0.1 and 0.01 because the effect of bacterial density on callus infection time is a key effect on transgenic efficiency, and excess proliferation of *Agrobacterium* decreases the transformation frequency during co-cultivation step (Ozawa, 2009). Callus appearance differed little between the two *Agrobacterium* concentrations (Figures 5C–F), except that calli were slightly browner at OD 0.1 during the first 2 weeks in the first selection media (Figure 5D). After selection steps, calli were transferred to MSre-CH50 (MS regeneration media supplemented with 500 mg l^−1^ claforan and 50 mg l^−1^ hygromycin) for ∼1.0 month (Figures 5G,H). However, no green spots appeared on calli from MSre-CH50 media 45 DAT. After transferring calli onto MS-C media, many green spots appeared and callus shooting occurred (Figure 5I). Four regenerated plants were obtained and subjected to acclimation for 10 days to produce hardier seedlings (Figure 5J). Subsequently, T_0_ plants were grown in a greenhouse to check for inserted genes and to obtain T_1_ seeds for further analyses (Figure 5K).

### Inserted gene check by genomic PCR

**Figure 6:**
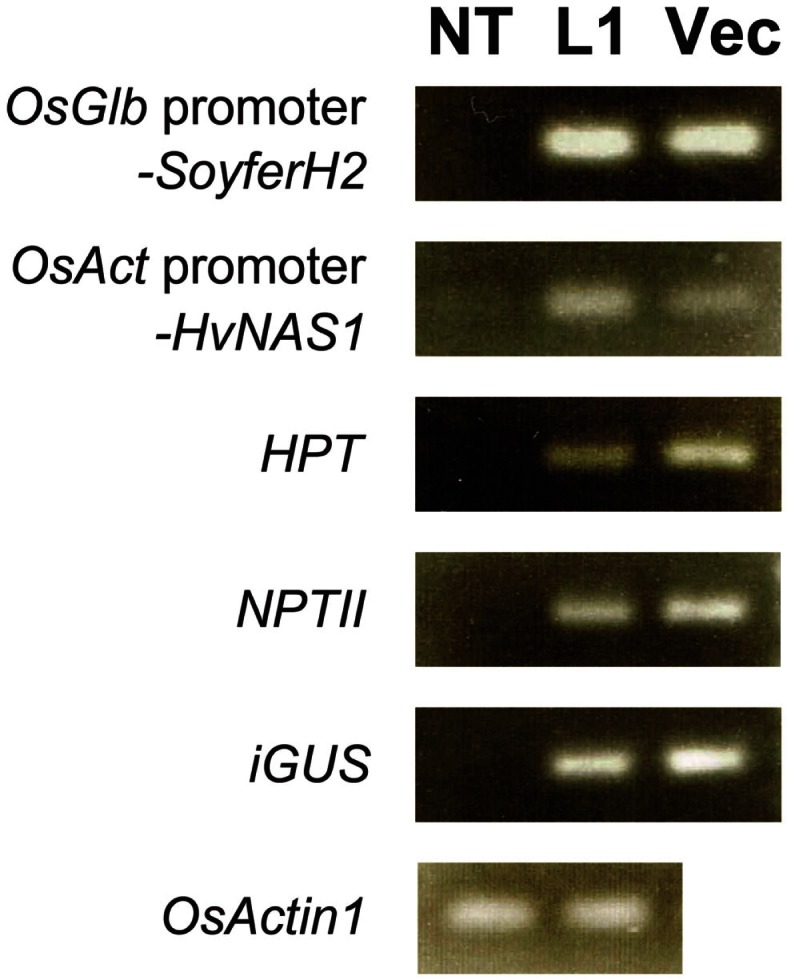
**Confirmation of gene insertion in transgenic Paw San Yin-Fer-NAS-YSL2 line 1**. NT, non-transgenic Paw San Yin line; L1, Paw San Yin-Fer-NAS-YSL2 line 1; Vec, Fer-NAS-YSL2 vector (positive control). *OsGlb* promoter-*SoyferH2*, soybean *Ferritin* gene *SoyferH2* with promoter region of the 26 kDa *OsGlb1* gene; *OsAct* promoter*-HvNAS1*, barley nicotianamine synthase 1 gene with promoter region of the rice *OsActin1* gene; *HPT*, hygromycin phosphotransferase gene; *NPTII*, neomycin phosphotransferase II gene; *iGUS*, β-glucuronidase gene *with an intron*; *OsActin1*, endogenous rice *actin* gene.

We checked four regenerated plants for selected gene insertions using genomic PCR. Paw San Yin-Fer-NAS-YSL2 line 1 (L1) contained insertions, including *OsGlb* promoter-*SoyferH2*, *OsAct* promoter-*HvNAS1*, *HPT*, *NPTII*, and *iGUS*, which was also the case in the Fer-NAS-YSL2 vector itself (Figure 6). The inserted genes were lacking in non-transgenic Paw San Yin (NT). We detected the endogenous sequence for the rice *OsActin1* gene in L1 and NT.

### Metal concentrations in T_1_ polished seeds

**Figure 7:**
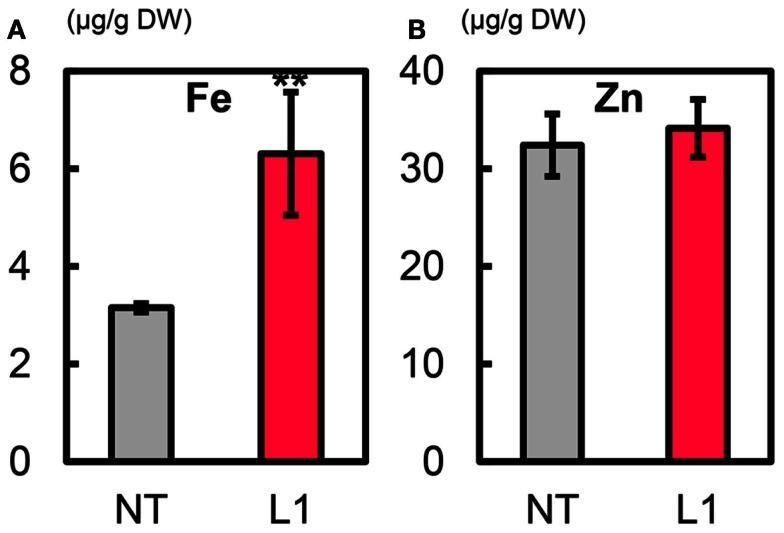
**Metal concentrations in T_1_ polished seeds of Paw San Yin-Fer-NAS-YSL2**. **(A)** Fe concentration. **(B)** Zn concentration. NT, non-transgenic Paw San Yin. L1, Paw San Yin-Fer-NAS-YSL2 transgenic line 1. Bars represent means ± SE, *n* = 3. Asterisks (**) above the bars indicate significant differences at *P* < 0.01 between NT and L1 (determined by*t*-test).

The mean Fe concentration in T_1_ polished seeds of L1 was 6.3 μg g^−1^, a value double that in NT (3.2 μg g^−1^) (Figure 7A). The mean Zn concentration in L1 seeds was ∼34.2 μg g^−1^, slightly higher than in NT seeds (32.4 μg g^−1^) (Figure 7B). L1 had a higher manganese (Mn) concentration than NT, its copper (Cu) concentration was similar to that of NT, and its calcium (Ca) concentration in polished seeds was lower than in NT (Figures S16A–C in Supplementary Material**)**. In T_1_ brown seeds, L1 had higher Fe and Mn concentrations and lower Cu concentration than NT (Figures S17A,D,E in Supplementary Material**)**. Zn and Ca concentrations in brown seeds were not different between L1 and NT (Figures S17B,C in Supplementary Material**)**. In T_1_ husk, L1 had lower Zn concentration and higher Ca and Cu concentrations than NT (Figures S18B–D in Supplementary Material**)**. The cadmium (Cd) concentration was lower in polished seeds, brown seeds and husk of L1 than in those of NT (Figures S16D, S17F, and S18E in Supplementary Material).

### Gene expression in immature T_2_ seeds

**Figure 8:**
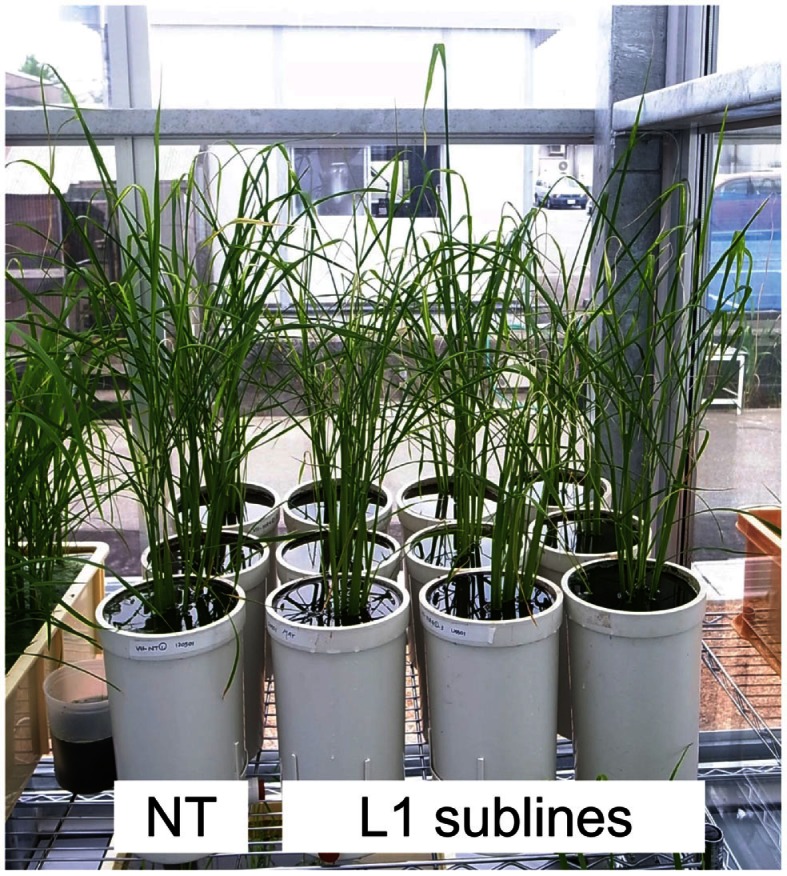
**Greenhouse-grown T_1_ plants**. Photograph was taken during tillering stage at 30 days after transplanting. NT, non-transgenic Paw San Yin. L1 sublines, Paw San Yin-Fer-NAS-YSL2 transgenic line 1 sublines.

**Figure 9:**
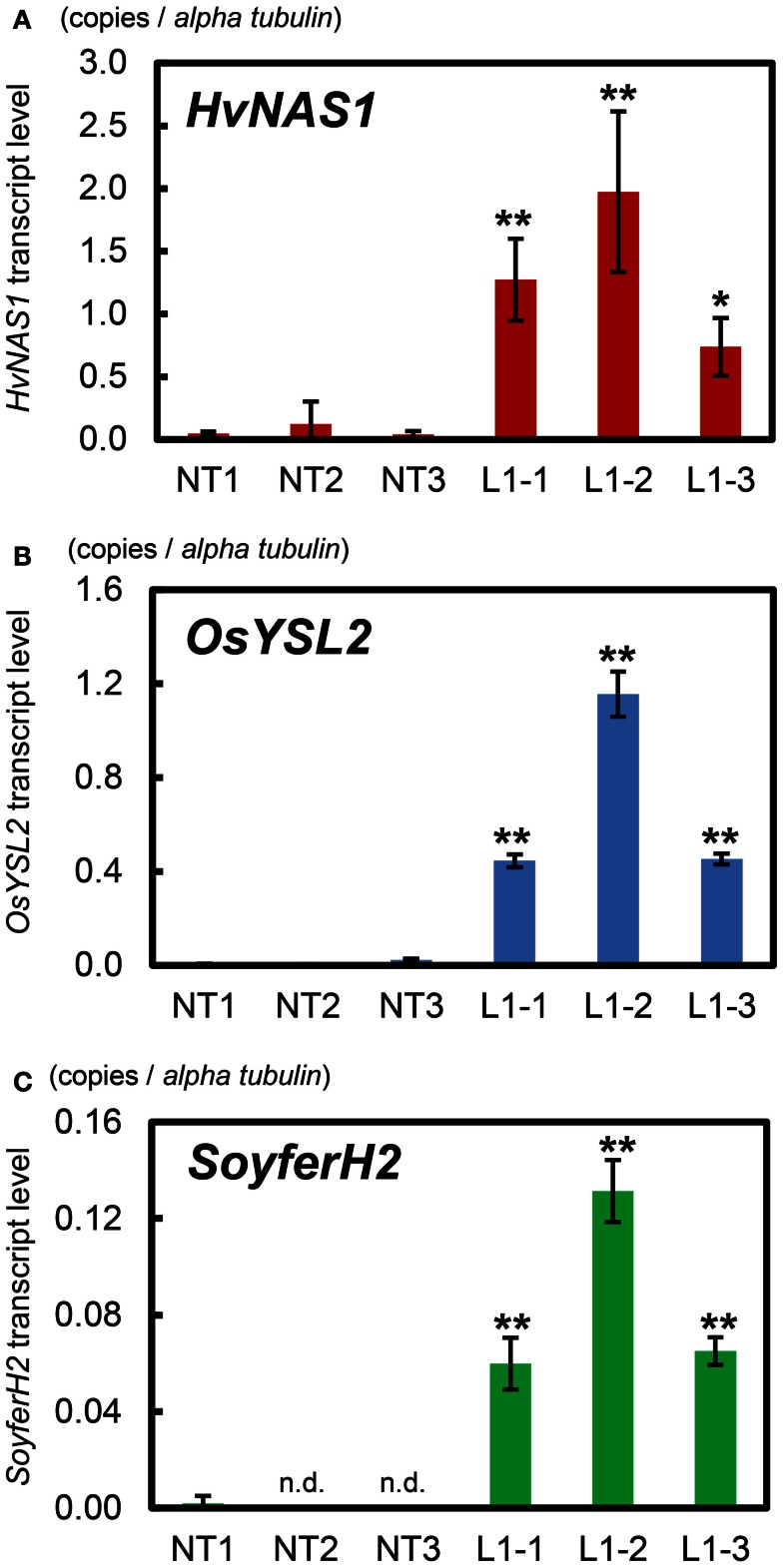
**Quantitative real-time RT-PCR analysis of *HvNAS1*, *OsYSL2*, and *SoyferH2***. **(A)** *HvNAS1*, **(B)** *OsYSL2*, and **(C)** *SoyferH2* expression levels. T_1_ plants were cultivated in commercial soil (Bonsolichigou) in a greenhouse. Total RNA was extracted from immature T_2_ seeds (seeds at the early milky stage, 10 days after fertilization) of each line (*n* = 3). NT, non-transgenic Paw San Yin. L1-1, L1-2, and L1-3, Paw San Yin-Fer-NAS-YSL2 transgenic line 1 sublines. Bars represent means ± SE of three independent real-time RT-PCR reactions. Asterisks (*) and (**) above the bars indicate significant differences at *P* < 0.05 and *P* < 0.01, respectively, between NT and L1 sublines (determined by *t*-test). n.d., not detected.

T_1_ seeds of L1 were germinated on MS-CH50 media. Subsequently, we cultivated T_1_ plants (sublines) in a greenhouse. No significant differences were observed in plant growth and morphology between L1 sublines and NT under these cultivation conditions (Figure 8). We confirmed the expressions of *HvNAS1*, *OsYSL2*, and *SoyferH2* in immature T_2_ seeds of L1 sublines by real-time RT-PCR analysis (Figure 9). Enhanced expressions of *HvNAS1*, *OsYSL2*, and *SoyferH2* genes were detected in L1 sublines compared with NT (Figure 9). *HvNAS1* expression in NT was barely detectable because sequences of *OsNAS1* and *HvNAS1* were very similar, and *OsNAS1* expression may be detectable in this line (Figure 9A). Expression of *OsYSL2* was very low in NT (Figure 9B). *SoyferH2* expression was undetectable in the NT line (Figure 9C). In contrast, *HvNAS1*, *OsYSL2*, and *SoyferH2* were strongly expressed in the L1-1, L1-2, and L1-3 sublines.

### Metal concentrations in T_2_ seeds

**Figure 10:**
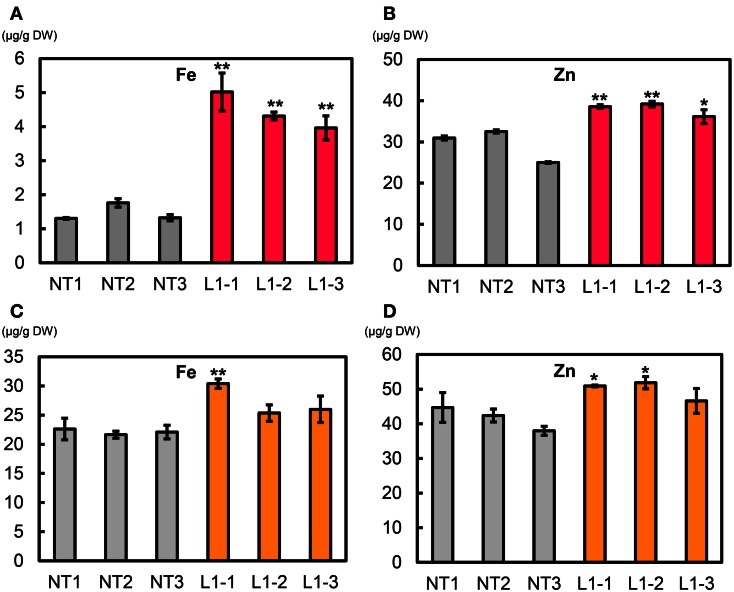
**Metal concentrations in T_2_ polished and brown seeds of Paw San Yin-Fer-NAS-YSL2**. **(A)** Fe concentration in T_2_ polished seeds. **(B)** Zn concentration in T_2_ polished seeds. **(C)** Fe concentration in T_2_ brown seeds. **(D)** Zn concentration in T_2_ brown seeds. NT, non-transgenic Paw San Yin. L1-1, L1-2, and L1-3, Paw San Yin-Fer-NAS-YSL2 transgenic line 1 sublines. Bars represent means ± SE, *n* = 3. Asterisks (*) and (**) above the bars indicate significant differences at *P* < 0.05 and *P* < 0.01, respectively, between NT and L1 sublines demonstrated by *t*-tests.

We measured metal concentrations in T_2_ polished seeds from the L1-1, L1-2, and L1-3 sublines and in NT (Figures 10A,B, Figure S19 in Supplementary Material). Mean Fe concentrations in polished seeds of L1-1, L1-2, and L1-3 sublines were 5.02, 4.31, and 3.96 μg g^−1^, respectively, values that were 3.0–3.4-fold higher than values in NT seeds (1.46 μg g^−1^) (Figure 10A). Mean Zn concentrations in polished seeds of L1-1, L1-2, and L1-3 sublines were 38.6, 39.2, and 36.1 μg g^−1^, respectively, values that were 22–33% higher than that in NT seeds (29.5 μg g^−1^) (Figure 10B). No clear differences were observed in polished seed concentrations of Ca, Mn, and Cu between the L1-1, L1-2, and L1-3 sublines and NT (Figures S19A–C in Supplementary Material). Polished seed Cd concentrations in the L1-1, L1-2, and L1-3 sublines were reduced by ∼20% compared to NT seeds (Figure S19D in Supplementary Material).

Mean T_2_ brown seed Fe concentrations in the L1-1, L1-2, and L1-3 sublines were 30.4, 25.4, and 26.0 μg g^−1^, respectively; these values were 15–37% higher than that in NT seeds (22.1 μg g^−1^) **(**Figure 10C). Mean T_2_ brown seed Zn concentrations in the L1-1, L1-2, and L1-3 sublines were 50.9, 51.9, and 46.6 μg g^−1^, respectively; these values were 12–24% higher than that in NT seeds (41.7 μg g^−1^) (Figure 10D). Ca, Mn, and Cu polished rice concentrations in the L1-1, L1-2, and L1-3 sublines were little different from that in NT (Figures S20A–C in Supplementary Material). Mean T_2_ brown seed Cd concentrations in the L1-1, L1-2, and L1-3 sublines were 0.07, 0.04, and 0.03 μg g^−1^, respectively; these values were two to fourfold lower than that in NT (0.1 μg g^−1^) (Figure S20D in Supplementary Material).

T_2_ husk Fe and Zn concentrations were similar between the L1 sublines and NT (Figures S21A,B in Supplementary Material). Ca, Mn, and Cu were higher in the sublines than in NT (Figures S21C–E in Supplementary Material).

### Fe and Zn contents per seed

**Figure 11:**
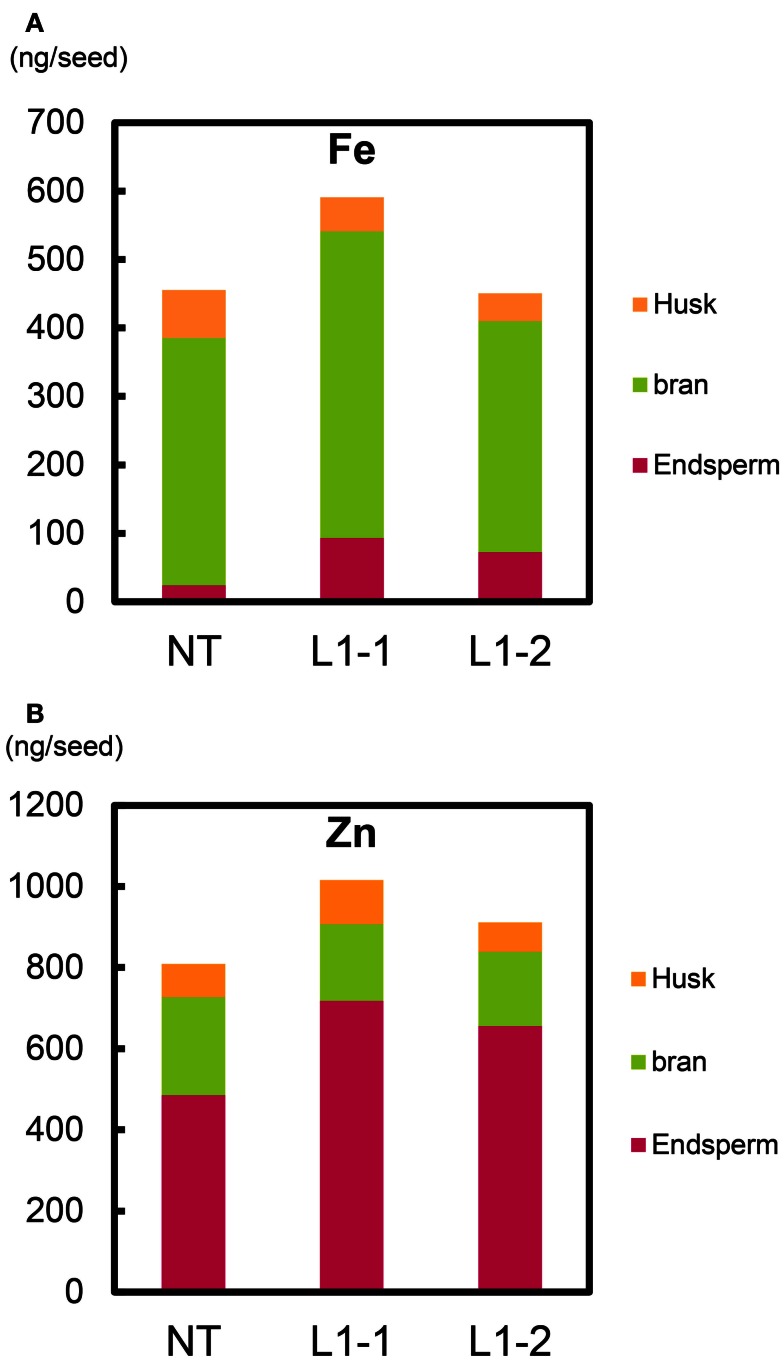
**Fe and Zn content per T_2_ seed of Paw San Yin-Fer-NAS-YSL2**. **(A)** Fe content in husk, bran, and endosperm per T_2_ seed. **(B)** Zn content in husk, bran, and endosperm per T_2_ seed. NT, non-transgenic Paw San Yin; L1-1 and L1-2, Paw San Yin-Fer-NAS-YSL2 transgenic line 1 sublines. Metal content of polished seed is shown as endosperm. Metal content of bran is calculated by subtracting metal content of polished seed from that of brown seed.

We estimated the partitioning of Fe and Zn among components of the seed structure (Figure 11, Figure S22 in Supplementary Material). Within T_2_ seeds of the L1-1 and L1-2 sublines, the total mean Fe contents were 590 and 450 ng seed^−1^ (including endosperm, bran, and husk), respectively; the value for NT was 455 ng seed^−1^ (Figure 11A). Mean Fe contents in endosperm (polished seed) of the L1-1 and L1-2 sublines (93.4 and 72.2 ng seed^−1^, respectively) were three to fourfold higher than that of NT (23.9 ng seed^−1^). Thus, the new lines had higher Fe allocation to edible seed portions than to either bran or husk (Figure 11A).

Total mean Zn contents per seed in the L1-1 and L1-2 sublines (1016 and 911 ng seed^−1^, respectively) were higher than in NT (808 ng seed^−1^) (Figure 11B). The endosperm Zn contents in the L1-1 and L1-2 sublines were 1.4–1.5-fold higher than in NT seeds. We observed similar trends in differences between the L1 line and NT in T_1_ seeds (Figure S22 in Supplementary Material).

## Discussion

### Paw San Yin was selected for rice transformation

Calli of Myanmar rice varieties were smaller than those of cultivar TK. The small, soft calli had limited tolerance to *Agrobacterium*-infection during transformation in our transgenic trials. However, callus inductions were good in V3, V6, and V14, and proliferations were closely similar to that of TK (Figure S4 in Supplementary Material). Particularly, V3 had a good callus induction response, and induced calli of V3 were largest; they were clear yellow and had the highest hardness index, traits that were closely similar to those of TK calli (Figure 1). These callus attributes make V3 suitable for rice transformation. *Agrobacterium*-infection is an important step in practical transformation, and callus tolerance of this infection is required for a successful outcome. Importantly, V3 calli in the N6D-N6D-MSre-MS medium combination maintained good condition in MS rooting media and produced many regenerated plantlets in the regeneration test (Figure 1A).

Myanmar people have one of the highest rice consumption rates in the world (FAO, 2002). The estimated polished seed Fe concentration needed to increase for these people is ∼8.6 μg g^−1^ (Supplementary Material). Our premise was that by using a high-Fe rice variety, we would be able to produce lines with much higher levels of Fe biofortification through transgenic transformations using the Fer-NAS-YSL2 vector (Masuda et al., 2012). Myanmar people will probably readily accept a novel Fe-biofortified rice produced from a native variety, and it was for this reason that we screened diverse Myanmar varieties for genetic variation in micronutrient concentrations (Aung et al., unpublished data). This screening identified Paw San Yin (V3) as a variety with high-Fe concentration (∼2.2 μg g^−1^) in relation to other popular rice strains tested (Aung et al., unpublished data): V3 had more than double the Fe content of the variety with the lowest concentration (Ma Naw Htun) (Aung et al., unpublished data) and double the content of field-grown TK rice (Masuda et al., 2012). Moreover, harvested Paw San Yin seed converts readily to a high-quality, aromatic rice product for human consumption. Grusak and Cakmak (2005) demonstrated that aromatic rice genotypes contain consistently more Fe and Zn than non-aromatic genotypes, indicating that the aromatic germplasm has promise as a genetic resource for improving rice micronutrient levels. Therefore, we selected Paw San Yin (V3) for practical transformation to produce Fe-biofortified rice.

### Transformation of Paw San Yin-Fer-NAS-YSL2

Our transformation procedure followed, except for a few modifications, an established protocol for *japonica* variety cv. TK developed by Hiei et al. (1994) and Akiyama et al. (1997). Hiei et al. (1994) reported that prior to infection, pre-cultivation of calli on fresh medium for 4 days is an important first step in *japonica* rice transformation. We demonstrated that a longer 8-day pre-incubation period was required for production of hard V3 calli for transformation. Callus condition was good during induction and *Agrobacterium* co-cultivation, though calli size were smaller than those of TK (Figures 5A–C).

Transformation efficiency was low in Paw San Yin-Fer-NAS-YSL2. Two possible explanations exist for this outcome. Although Paw San Yin calli were in adequate condition for transformation, they were negatively affected and damaged by *Agrobacterium*-infection to a greater extent than TK calli, where *Agrobacterium* presence was minimal in calli after the *Agrobacterium* wash (**data not shown**). Therefore, low regeneration rates from selected calli in this Paw San Yin variety may have resulted from adverse effects of *Agrobacterium*-infection when the bacterium further propagated on calli during the selection periods (Figures 5D–F). Hence, we applied a claforan concentration double that was used for TK transformation when selecting media that prevented *Agrobacterium* propagation. Green spots did not appear on MSre-CH50 through 1 month following transfer. The large size of the vector that we introduced may also have been responsible for the low rate of transformation we obtained (Masuda et al., 2012) (Figure S2 in Supplementary Material).

### Gene expression analysis and functions of introduced genes

Real-time RT-PCR analyses confirmed elevated expressions of *HvNAS1*, *OsYSL2*, and *SoyferH2* transgenes in Paw San Yin-Fer-NAS-YSL2 L1-1, L1-2, and L1-3 T_2_ sublines (Figure 9). *HvNAS1* encodes barley nicotianamine synthase, which biosynthesizes the divalent metal chelator nicotianamine (Higuchi et al., 1999), and this gene was overexpressed by the constitutive rice *Actin1* promoter in Fer-NAS-YSL2 vector (Figure S2 in Supplementary Material) (Masuda et al., 2012). As is the case for TK transformants with introduced Fer-NAS-YSL2 (Masuda et al., 2012), *HvNAS1* was strongly expressed in Paw San Yin-Fer-NAS-YSL2 T_2_ seeds during the seed milky stage 10 days after fertilization (early seed maturation stage) (Figure 9A). *OsYSL2* encodes an Fe(II)-nicotianamine and Mn(II)-nicotianamine transporter, which are responsible for internal metal transport in rice (Koike et al., 2004; Ishimaru et al., 2010). The Fer-NAS-YSL2 vector contains two cassettes for *OsYSL2* expression (Figure S2 in Supplementary Material), each driven by an *OsSUT1* promoter that drives expression in the early seed maturation stage beginning 7 days after fertilization (Hirose et al., 2002) and by an endosperm-specific *OsGlb1* promoter (Qu and Takaiwa, 2004). As a result, *OsYSL2* was strongly expressed in Paw San Yin-Fer-NAS-YSL2 T_2_ seeds during the seed maturation stage 10 days after fertilization (Figure 9B). *SoyferH2* encodes the soybean Fe storage protein ferritin, which was driven by endosperm-specific *OsGlb1* and *OsGluB1* promoters in the Fer-NAS-YSL2 vector (Figure S2 in Supplementary Material). Qu and Takaiwa (2004) demonstrated that the GUS reporter protein accumulates in the early maturation stage 7 days after fertilization in *OsGlb1* promoter-*GUS* and 2.3-kb *OsGluB1* promoter-*GUS* plants. Hence, it was assumed that *ferritin* expression under the control of the *OsGlb1* and 2.3-kb *OsGluB1* also initiates ferritin protein accumulation in the early seed maturation stage 7 days after fertilization. We clearly detected *SoyferH2* expression in Paw San Yin-Fer-NAS-YSL2 10 days after fertilization (Figure 9C). Thus, we assumed that coordination occurred in the timing of enhancement in nicotianamine production through *HvNAS1* overexpression, the enhancement of Fe(II)-nicotianamine transport by *OsYSL2* expressed under the control of the *OsSUT1* and *OsGlb1* promoters, and the enhancement of Fe accumulation by the *OsGlb1* promoter-*ferritin* and 2.3-kb *OsGluB1* promoter-*ferritin* (Masuda et al., 2012).

Fe contents in polished seeds of Paw San Yin-Fer-NAS-YSL2 sublines were three to fourfold higher than those of NT (Figure 11A). We supposed that Fe transport into the plant body and into grain was enhanced by elevated production of nicotianamine by overexpression of *HvNAS1* (Lee et al., 2009; Masuda et al., 2009a; Johnson et al., 2011) and improved expression of the *OsSUT1 promoter-OsYSL2* (Koike et al., 2004; Ishimaru et al., 2010). Moreover, the Fe storage protein ferritin, which accumulated in the endosperm, may have worked cooperatively in Fe translocation to the endosperm component of grain. We demonstrated that genes involved in Fe transport and accumulation in the endosperm (*HvNAS1*, *OsYSL2*, and *SoyferH2*) work efficiently together to increase Fe content in the endosperm, the edible component of the rice grain.

### Seed Fe concentration increased in Paw San Yin-Fer-NAS-YSL2

Fe concentration in Paw San Yin-Fer-NAS-YSL2 brown seed was 15–37% higher than that in NT brown seed (Figure 10C) and 3.4-fold higher in polished grain (Figure 10A), clearly demonstrating that Fe was effectively translocated into the endosperm. There were reports of other high-Fe-content rice, showing two to over three times increase in Fe concentration of various rice varieties (Goto et al., 1999; Lucca et al., 2001; Vasconcelos et al., 2003; Lee et al., 2009; Masuda et al., 2009a; Johnson et al., 2011; Paul et al., 2012). Using the transgenic approaches, most trials reported the rice varieties efficient for *in vitro* and molecular research, or the varieties which have already established transgenic procedures. There are still only a few trials working with popular rice varieties for Fe biofortification. In our present research, we generated the Fe biofortified Paw San Yin rice, which is a high-quality and currently cultivated and consumed rice variety in Myanmar, with 3.4-fold increase in Fe concentration in rice endosperm.

We found that the transgenic efficiency of Paw San Yin was low and difficult to obtain many transgenic lines. The main limiting factor to increase in Fe-density of the transgenic Paw San Yin is the difficulty in obtaining many transgenic lines which include all inserted genes. In our previous report by Masuda et al. (2012), the line with six times increase in Fe concentration of TK rice was selected among 45 lines. If many more lines of Paw San Yin were obtained, there is a possibility to obtain higher Fe lines.

In the present study, our objective was to increase the polished rice Fe concentration about 4.5-fold in the high Fe variety, Paw San Yin, based on a per capita rice consumption of 578 g day^−1^ in Myanmar (Supplementary Material). T_2_ seed analysis demonstrated that the polished seed Fe concentration was 3.4-fold higher than in NT line (Figure 10A). Thus, we closely approached the estimated target level. Therefore, it can be assumed that 3.4-fold increase in Fe concentration of Paw San Yin variety is remarkable.

Fer-NAS-YSL2 gene improved Fe accumulation and transport, but Fe uptake was not enhanced yet. Mugineic acid is known as a natural Fe(III) chelator used in Fe acquisition from the rhizosphere in barley and some other graminaceous plants. Masuda et al. (2008) analyzed the transgenic rice line carrying barley *IDS3* gene, which is the mugineic acid synthase gene. The transformants showed 1.4-fold increase in Fe concentration in polished seeds of rice grown in field cultivation. If barley *IDS3* is introduced to this Fer-NAS-YSL2 gene to enhance Fe uptake from soil, there is a possibility to increase more Fe accumulation in grain.

### Zn concentration increased in Paw San Yin-Fer-NAS-YSL2

Zn deficiency is a serious problem in Myanmar (Hotz and Brown, 2004). In polished seeds produced from rice grown in the MRRC field, Paw San Yin had the highest Zn concentration (∼19.1 μg g^−1^), which was almost double those in a range of other popular rice varieties (Aung et al., unpublished data**)**. The Zn concentration in polished seeds was elevated by 30% in Paw San Yin-Fer-NAS-YSL2 sublines (Figure 10B), which may meet the target concentration for Zn biofortification in the Myanmar diet.

Zn content in the endosperm of Paw San Yin-Fer-NAS-YSL2 was 1.4-fold higher than that in NT endosperm (Figure 11B). This outcome may have resulted from *HvNAS1* overexpression and endosperm-specific expression of the *ferritin* gene. *NAS* overexpression increases both Fe and Zn concentrations in seeds (Lee et al., 2009; Masuda et al., 2009a; Johnson et al., 2011). Endosperm-specific expression of *ferritin* also increases Zn concentrations in seeds (Goto et al., 1999; Vasconcelos et al., 2003). In comparison with Fe, Zn levels changed little, likely because more Zn occurs naturally in endosperm than in bran; hence, polishing has less of an effect on Zn levels than on Fe levels (Figure 11).

### Other metal concentrations in seeds of Paw San Yin-Fer-NAS-YSL2

Concentrations of other metals in both polished and brown seeds, including Ca, Mn, and Cu, were little different between Paw San Yin-Fer-NAS-YSL2 sublines and NT, although levels were somewhat higher in husks of transformed sublines (Figures S19A–C, S20A–C, and S21C–E in Supplementary Material). This lack of an effect may be attributable to specific targeting of the Fer-NAS-YSL2 vector toward accumulation of an elevated Fe concentration in endosperm. Thus, the Fer-NAS-YSL2 construct increased Fe concentrations in grain without reducing concentrations of other required minerals.

Cadmium is a toxic metal present in soil. It was detected in seeds of both NT and the Fer-NAS-YSL2 line (Figures S16D, S17F, S18E, S19D, and S20D in Supplementary Material). Cd concentrations in our samples were low compared to the level at which the metal becomes toxic in polished rice (∼0.4 μg g^−1^) (CODEX, 2012). Notably, Cd concentrations in brown and polished seeds of Paw San Yin-Fer-NAS-YSL2 T_1_ and T_2_ generations were remarkably lower than levels in Paw San Yin-NT (Figures S16D, S17F, S18E, S19D, and S20D in Supplementary Material). The Cd concentration in T_2_ polished seeds was about 20% lower in Paw San Yin-Fer-NAS-YSL2 than in NT (Figure S19D in Supplementary Material). One may reasonably argue that when Fe transportation to seed was increased in this transgenic rice, the expression levels of Fe transporters that also transport Cd, such as *OsIRT1* (Nakanishi et al., 2006), *OsNRAMP5* (Ishimaru et al., 2012; Sasaki et al., 2012), or other unknown Fe and Cd transporters, may decrease. Thus, it was assumed that Cd concentration in Paw San Yin-Fer-NAS-YSL2 seeds was lowered in the transgenic sublines. The seed Cd concentration in the *HvNAS1* overexpression line also decreased (Masuda et al., 2012). Hence, rice with an Fe content elevated through insertion of the Fer-NAS-YSL2 construct will likely be useful in reducing dietary Cd levels, especially in crops grown on Cd contaminated soil.

## Conclusion

Our starting premise was that currently consumed rice varieties with high Fe and Zn contents would be suitable starting material for the production of Fe-fortified grain. We screened methods for regenerating diverse Myanmar rice varieties and identified those varieties with good callus induction and high regeneration efficiency, which might contribute to transgenic development.

Paw San Yin is a high-quality, popular rice variety. The first Myanmar transgenic rice was produced successfully from this variety. Through our program, we achieved Fe-biofortified Paw San Yin rice with an Fe concentration 3.4-fold higher than the concentration in control seeds. The degree of Fe increase closely approached the dietary target for Myanmar people. The Zn concentration in the new transgenic sublines may also fulfill Zn requirements for the Myanmar population.

## Conflict of Interest Statement

The authors declare that the research was conducted in the absence of any commercial or financial relationships that could be construed as a potential conflict of interest.

## Supplementary Material

The Supplementary Material for this article can be found online at: http://www.frontiersin.org/Plant_Physiology/10.3389/fpls.2013.00158/abstract
