# Supplementary material for: Iron Biofortification of Myanmar Rice
Source: Front Plant Sci. 2013 May 27;4:158. doi: 10.3389/fpls.2013.00158 (PMC3664312; doi:10.3389/fpls.2013.00158)
Supplement: Supplementary file 1 [file 47646_Nishizawa_DataSheet1.PDF]

Supplementary Material of

Iron biofortification of Myanmar rice

May Sann Aung[1,2†], Hiroshi Masuda[2†], Takanori Kobayashi[2], Hiromi Nakanishi[1], Takashi Yamakawa[1] and Naoko K. Nishizawa[1, 2*]

1) Laboratory of Plant Biotechnology, Department of Global Agricultural Sciences, Graduate School of Agricultural and Life Sciences, The University of Tokyo, Bunkyo-ku, Tokyo, Japan
2) Laboratory of Plant Cell Technology, Research Institute for Bioresources and Biotechnology, Ishikawa Prefectural University, Nonoichi, Ishikawa, Japan

†These authors contributed equally to this work.

[*]Correspondence:

Dr. Naoko K. Nishizawa
Laboratory of Plant Cell Technology
Research Institute for Bioresources and Biotechnology
Ishikawa Prefectural University, 1-308 Suematsu,
Nonoichi, Ishikawa, 921-8836, Japan.
Tel / Fax: +81 76 227 7505
Email: annaoko@mail.ecc.u-tokyo.ac.jp

SUPPLEMENTARY RESULTS AND DISCUSSION

CALLUS INDUCTION AND CALLUS GROWTH TESTS UNDER STANDARD 2,4-D CONCENTRATIONS

Use of actively growing embryogenic calli contributes importantly to efficient transformation (Hiei et al., 1997, Masuda, 2009b). Thus, before producing high-Fe Myanmar rice, we performed analyses of callus induction and growth in diverse Myanmar rice varieties; no previous information existed on Myanmar varieties or medium types suited to the production of transgenic lines in real transformations.

N6 medium is widely used for callus tissue culture in many plants (Chu, 1978; Tabei, 2012). Both 2N6 and N6D medium are modified from N6 medium and are used for rice transformation. N6D medium induces better calli in the Tsukinohikari variety than 2N6 medium (Masuda, 2009b). Therefore, we expected differences in callus induction between 2N6 and N6D media in Myanmar rice varieties, and we tested both media for callus induction.

Fifteen Myanmar rice varieties were used in the regeneration trials. Calli were induced on N6D and 2N6 media in various medium combinations. Calli were initiated from the basal (mesocotyl or coleoptile) region of germinated seeds after cultivation for 1 week. Proliferation of calli continued through the fourth week of culture. Callus induction capacity in some Myanmar rice varieties, especially V3 and V14 on N6D media and V11 and V15 on both media, matched performance of the variety Tsukinohikari (**Supplementary Figure 3**). However, induction performance in all other Myanmar rice varieties was worse than in Tsukinohikari, especially on 2N6 media. In some Myanmar rice varieties, such as V1, V2, V4, V5, V7, V9, and V12, calli did not proliferate well in either culture medium even 27 DAG (**Supplementary Figure 3**).

Longer roots were particularly frequent in varieties with low callus induction capacity (**Supplementary Figure 3**).

Differences in the extent of callus growth became more marked when calli were transferred to new medium for subsequent growth. Callus formation (number of calli) and callus quality (size, yellowness, and hardness) varied greatly on different media 19 DAT, even within the same variety (**Supplementary Figure 4**). Compared to Tsukinohikari, some of the Myanmar rice varieties had low rates of callus proliferation on both 2N6-2N6 and N6D-N6D media. Callus formations by V1, V7 and V13 were extremely low on 2N6 media (**Supplementary Figure 4**). In contrast, some varieties, such as V3, V6, V11, V14, and V15, had higher callus proliferation rates, particularly on N6D-N6D culture media (**Supplementary Figure 4**). Since the degree of callus formation and callus quality varied greatly within and among varieties, callus growth was dependent on the culture response of each variety.

The callus induction and growth rate were remarkably different between 2N6 and N6D culture media and among varieties (**Supplementary Figure 3,4**). In many cases, N6D media yielded good calli and enhanced callus induction. This superior performance may have resulted from the proline content in N6D media (see "**MEDIA USED FOR CALLUS INDUCTION AND REGENERATION TEST**" in the "MATERIALS AND METHODS" for references). Proline is an amino acid that provides protection against stress through a diversity of mechanisms (Hare and Cress, 1997; Nanjo et al., 1999; Toki et al., 2006; Sivakumar et al., 2010), and it may contribute to the recovery of viable embryogenic calli following various *in vitro* stresses. Moreover, proline is used as a precursor in the processes of metabolism and cell division (Santos et al., 1996), and it is most efficient in callus promotion (Thadavong et al., 2002). Raval and Chattoo (1993) demonstrated that calli induced on N6 medium supplemented with proline maintain regeneration potential for a longer time than calli

on other media. Furthermore, the high content of Gellan Gum in N6D media forms a hard substrate that promotes good callus formation.

REGENERATION TEST FOLLOWING CALLUS INDUCTION IN STANDARD 2,4-D CONCENTRATIONS

We observed the regeneration efficiency of 15 Myanmar rice varieties in various medium combinations. High-quality calli of Ayar Min (V2) were readily induced on N6D-N6D; many green spots appeared on the calli 11 DAT (**Supplementary Figure 5**). After transfer to MS media 12 DAT, many regenerated shoots and plants formed. Regeneration efficiency was moderate in this variety. No plants regenerated in the 2N6-2N6 treatment. Calli of similar condition were produced by the Shwe War Htun (V4) high-yield variety (**Supplementary Figure 6**). Callus induction and growth were elevated on N6D-N6D, and regenerated plants grew. 2N6-2N6-derived calli did not produce regenerated plants. The high-quality variety Yezin Lone Thwe (V6) had elevated callus formation capacity 34 DAG and had extensive proliferations 19 DAT, especially on N6D-N6D (**Supplementary Figure 7**). Green spots appeared on calli grown on N6D-N6D after transfer to MSre media; a few regenerated plants were obtained on MS media.

Responses of the Sin Nwe Yin (V9) high-yield variety differed widely between the two medium combinations (**Supplementary Figure 8**). Callus inductions (34 DAG) were poor on both media types. However, 12 DAT callus condition was better on N6D medium than on 2N6 medium. When calli were subsequently transferred to MSre media, N6D-N6D-derived calli performed well and produced green shoots earlier than most other varieties. Callus induction was moderate and regeneration rate low in the Hmawbi 4 (V13) high-quality variety grown on N6D-N6D media (**Supplementary Figure 9**). Ayar Min (V2), Shwe War Htun (V4), Yezin Lone Thwe (V6), Sin Nwe Yin (V9), and

Hmawbi 4 (V13) were not able to regenerate on 2N6-2N6 (**Supplementary Figures 5–9**). Callus induction and growth of the Hmawbi 5 high-yield variety (V14) were better on N6D-N6D to than on 2N6-2N6 (**Supplementary Figure 10**), but callus condition was poor on MSre, and few regenerated plants were obtained (**Supplementary Figure 10A**). On the other hand, 2N6-2N6 showed low callus induction and growth but elevated regeneration efficiency occurred on MSre and MS media (**Supplementary Figure 10B**). In general, the N6D-N6D-MSre-MS medium combination promoted the best performance in most Myanmar rice varieties.

Callus conditions of V2 and V3 were good on MS rooting media, and many plantlets regenerated in the N6D-N6D-MSre-MS medium combination (**Figure 1, Supplementary Figure 5**). Although many green spots appeared on MS, few regenerated plants were produced by Yezin Lone Thwe (V6), for example (**Supplementary Figure 7A**), and none by Kyaw Zay Ya (V1) (**Supplementary Figure 11C**) or Thee Htet Yin (V8) (**Supplementary Figure 12D**). Thus modification of MS media composition will be required for optimum regeneration in some Myanmar rice varieties.

When callus condition was poor on MSre or MS media, regeneration efficiency was low or zero, even when callus induction and growth were good (**Supplementary Figures 7A, 10A, 13C**). However, when callus condition was superior on either MSre or MS media, regeneration efficiency was high even when callus induction and growth were poor (**Supplementary Figure 5A**).

EFFECTS OF 2,4-D CONCENTRATION ON CALLUS INDUCTION AND REGENERATION ON N6D AND 2N6 MEDIA

Callus inductions on 2N6 or N6D media were inadequate in some Myanmar rice varieties. Thus, in Kyaw Zay Ya (V1) and Yadana Toe (V10), callus induction was

inferior and no regenerated plants were produced on either N6D-N6D-MSre-MS or 2N6-2N6-MSre-MS medium combinations (**Supplementary Figures 11, 13**). Thee Htet Yin (V8) and Hmawbi 2 (V11) produced only small numbers of regenerated plants, suggesting that improvement of callus induction should be a future research objective (**Supplementary Figures 12, 14**).

2,4-D operates as a synthetic auxin plant hormone, and optimizing its concentration is important for production of superior calli for transformation (Bajaj, 1991). Pandey et al. (1994), Thadavong et al. (2002), Abeyaratne et al. (2004), and Summart et al. (2008) demonstrated that a 2,4-D concentration of 2 mg l^{-1} promotes the best callus induction from mature rice seeds. Our analyses supported this conclusion for some Myanmar rice varieties. However, the 2,4-D concentration needed was genotype-dependent. Callus induction was inferior in V1, V5, V7, V8 and V10 on both N6D and 2N6 media and V11 on 2N6 medium containing 2 mg l^{-1} 2,4-D (**Supplementary Figures 11–14**). Thus, we tested the effects of 2,4-D at concentrations of 4 and 6 mg l^{-1} on callus induction and growth in some varieties; our aim was to improve callus induction and regeneration efficiency.

Media containing 6 mg l^{-1} of 2,4-D enhanced the efficiency of callus formation and regeneration in Kyaw Zay Ya (V1) (**Supplementary Figure 11**). Regeneration efficiencies of Thee Htet Yin (V8) and Yadana Toe (V10) were better in 4 mg l^{-1} 2,4-D than in 2 or 6 mg l^{-1} 2,4-D (**Supplementary Figures 12, 13**). Callus induction in Hmawbi 2 (V11) was superior at high 2,4-D concentrations in N6D media (**Supplementary Figure 14**). Improved callus induction enhanced the regeneration rates in these varieties. Moreover, regenerated plants were obtained in all varieties whose calli did not produce regenerated plants in both N6D and 2N6 media with 2 mg l^{-1} of 2,4-D concentrations. This result is concordant with findings of Sivakumar et al. (2010)

and Rajesh et al. (2008), who demonstrated that increased application of 2,4-D induces high rates of callus induction in *indica* genotypes.

Responses to increased 2,4-D concentration were variety-dependent. A concentration of 4 mg l^{-1} was best for V8 and V10, while 6 mg l^{-1} was best for V1 and V11 (**Supplementary Figures 11–14**). For the other Myanmar varieties tested, 2 mg l^{-1} was best (**Figure 2; Supplementary Table 1**). This combination of results agrees with findings of Raina (1989), who reported that 2,4-D is the most appropriate auxin for rice callus induction in tissue culture; however, as noted above, optimum concentrations of 2,4-D vary by plant source and genotype. Thus, testing each *indica* variety with a range of 2,4-D concentrations will help identify optimum media.

IDENTIFICATION OF BEST MEDIUM COMBINATIONS AND VARIETIES WITH SUPERIOR ATTRIBUTES AMONG MYANMAR RICE GENOTYPES

We determined callus quantity (numbers of calli induced) and quality (yellowness, size, and hardness) during callus induction and growth. We identified minimal values of callus size, hardness, and yellowness that were crucial for initiation of plant regeneration (**Supplementary Figure 15**) and its further progress. Current MSre and MS medium components were appropriate for varieties with scores ≥5 in hardness, yellowness and size. However, the regeneration efficiencies of some Myanmar rice varieties were low even when callus condition scores were high. In these cases, MSre and MS medium components should be modified in future for better performance.

By using diverse media, we identified varieties with good callus induction and high regeneration efficiency for further transformation. The best medium combination was also identified for each variety (**Figure 2; Supplementary Table 1**). Regenerated plants were obtained from many varieties. Among the 15 varieties tested, 13 varieties were able to regenerate (**Figure 3; Supplementary Table 1**). These 13 varieties may be

candidates for transgenic transformation using *Agrobacterium* in the best medium combinations we identified. Regeneration efficiency was very different among varieties (**Figure 3**). Most Myanmar rice varieties regenerated faster and more prolifically than Tsukinohikari. Sin Thwe Lat (V5) and Tu Kha Yin (V7) had a very low callus induction rate and no regenerated plants were obtained (**Figure 3; Supplementary Table 1**). Media composition for these two varieties will require future development.

The duration of time to green spot appearance was shorter in many Myanmar varieties than in Tsukinohikari (**Figure 4**). Time to green spot appearance is a crucial consideration for rice transformation. Shorter times to green spot appearance may enable transformation to plantlets before the finite life spans of the calli have run their course. In our regeneration trials, many calli gradually became brown, and regeneration efficiency dropped when calli were held for protracted periods on the medium (**data not shown**).

FERRITIN GENE

In the current study, we chose *SoyferH2* rather than *SoyferH1* as the soybean *ferritin* gene for expression in rice endosperm (**Supplementary Figure 2**). *SoyferH1* is readily digested by proteases, likely altering its structure and allowing Fe release; *SoyferH2* is more resistant to protease digestion (Masuda et al., 2001), and we judged it to be more appropriate for Fe accumulation in rice seeds and hence the production of high-Fe rice.

TARGET FE CONCENTRATION IN MYANMAR RICE FOR THE MYANMAR HUMAN POPULATION

Myanmar people are among the highest consumers of rice in Southeast Asia. Fe intake per person is 7.5–10.3 mg d^{-1} (FAO, 2002), but the Fe requirement for adult women is 15–18 mg d^{-1} (Institute of Medicine, 2001). The per capita shortfall is therefore ~5 mg

d^{-1}. Our objective is to bridge this shortfall by providing high-Fe rice. Therefore, Fe concentration needed to increase more in polished seeds is about 8.6 µg g^{-1} based on a per capita rice consumption of 578 g d^{-1} in Myanmar (Kennedy et al., 2002; Maclean et al., 2002). The polished seed Fe concentration of Myanmar rice varieties is around 1-2.5 µg g^{-1} (Aung et al., **unpublished data**). Thus, target Fe concentration required in polished seeds is about 9.5-11 µg g^{-1}.

The mean Fe concentration in Paw San Yin polished seeds produced at MRRC field is 2.2 µg g^{-1} DW (Aung et al., **unpublished data**). Thus, the Fe content of this variety should be about 4.5 times by Fe-biofortification to reach the target Fe-concentration level for the Myanmar population.

SUPPLEMENTARY REFERENCES

Abeyaratne, W. M., De Silva, U. N., Kumari, H. M. P. S., and Abeysiriwardena, D. S. De Z. (2004). Callus Induction, Plantlet Regeneration and Occurrence of Somaclonal Variation in Somatic Tissues of Some Indica Rice Varieties. *Annals of the Sri Lanka Department of Agriculture.* 6, 1-11.

Chu, C. C. (1978). The N6 medium and its applications to anther culture of cereal crops. in *Proceedings of Symposium on Plant Tissue Culture*, (Beijing: Science Press), 43-50.

Institute of Medicine. (2001) Food and Nutrition Board. Dietary Reference Intakes for Vitamin A, Vitamin K, Arsenic, Boron, Chromium, Copper, Iodine, Iron, Manganese, Molybdenum, Nickel, Silicon, Vanadium and Zinc. Washington, DC: National Academy Press.

Hare, P. D., and Cress, W. A. (1997). Metabolic implications of stress induced proline accumulation in plants. *Plant Growth Regul.* 21, 79-102.

Kawasaki. S. (2003). New high capacity binary shuttle vector. *United States Patent Application Publication* US 2003/0003585 A1.

Masuda, H. (2009b). Production of mineral biofortified rice. The University of Tokyo. Doctoral course thesis (in Japanese).

Masuda, T., Goto, F., and Yoshihara, T. (2001) A novel plant ferritin subunit from soybean that is related to a mechanism in iron release. *J. Biol. Chem.* **276**, 19575-19579.

Nanjo, T., Kobayashi, M., Yoshida, Y., Sanda, Y., Wada, K., Tsukaya, H., Kakubari, Y., Aamaguchi-Shinonaki, K., and Shinozaki, K. (1999). Biological functions of proline morphogenesis and osmotolerance revealed in antisense transgenic *Arabidopsis thaliana*. *Plant J.* 18, 185-193.

Pandey, S. K., Ramesh, B., and Gupta, P. K. (1994). Study on effect on genotype and culture medium on callus formation and plant regeneration in rice (*Oryza sativa* L.). *Indian J. Genet.* 54, 293-299.

Raina, S. K. (1989). Tissue Culture in Rice Improvement: Status and Potential. *Adv. Agron.*42, 339-398.

Raval, M., and Chattoo, B. B. (1993). Role of media constitutes and proline in callus growth, somatic embryogenesis and regeneration of *Oryza sativa* cv indica. *Indian J. Exp. Biol.* 31, 600-603.

Santos, M. A., Camara, T., Rodriguez, P., Claparols, I., and Torne, J. M. (1996). Influence of exogenous proline on embryogenic and organogenic maize callus subjected to salt stress. Plant Cell, Tissue and Organ Cult. 47, 59-65.

Tabei., Y. (ed). 2012. Transformation Protocol. Plants. Tokyo:Kagaku-Doujin. (in Japanese).

Thadavong, S., Sripichitt, P., Wongyai, W., and Jompuk, P. (2002). Callus induction and plant regeneration from mature embryos of glutinous rice (*Oryza sativa* L.) cultivar TDK1. *Kasetsart J. Nat. Sci.* 36, 334-344.

Toki, S., Hara, N., Ono, K., Onodera, H., Tagiri, A., Oka, S., and Tanaka, H. (2006). Early infection of scutellum tissue with *Agrobacterium* allows high-speed transformation of rice. *The Plant J.* 4, 969-976.

Supplementary Table 1 | Best medium combination tested for each variety, duration in each medium and number of regenerated plants

Variety number	Variety name	Medium combination and duration in each medium	Number of regenerated plants
**V1**	Kyaw Zay Ya	N6D6– N6D6–MSre–MS– Acclimation<br>31d 14d 13d 13d 3d	87
**V2**	Ayar Min	N6D – N6D – MSre – MS – Acclimation<br>28d 22d 7d 12d 3d	90
**V3**	Paw San Yin	N6D– N6D – MSre – MS – Acclimation<br>28d 9d 11d 17d 3d	47
**V4**	Shwe War Htun	N6D – N6D – MSre – MS – Acclimation<br>32d 11d 14d 17d 3d	58
**V5**	Sin Thwe Lat	N6D- N6D – MSre-MS<br>34d 19d 20d	0
**V6**	Yezin Lone Thwe	N6D – N6D – MSre – MS – Acclimation<br>33d 16d 11d 21d 3d	19
**V7**	Thu Kha Yin	N6D– N6D – MSre-MS<br>34d 19d 11d	0
**V8**	Thee Htet Yin	N6D4– N6D4–MSre–MS– Acclimation<br>31d 14d 13d 26d 3d	30
**V9**	Sin Nwe Yin	N6D – N6D – MSre – MS –Acclimation<br>34d 12d 17d 13d 3d	21
**V10**	Yadana Toe	N6D4– N6D4–MSre–MS– Acclimation<br>31d 14d 13d 26d 3d	21

Supplementary Table 1 (continued)

Variety number	Variety name	Medium combination and duration in each medium	Number of regenerated plants
**V11**	Hmawbi 2	N6D6– N6D6–MSre–MS– Acclimation<br>31d 11d 14d 17d 3d	85
**V12**	Hmawbi 3	N6D – 2N6 – MSre – MS – Acclimation<br>35d 18d 22d 15d 3d	17
**V13**	Hmawbi 4	N6D– N6D – MSre – MS – Acclimation<br>33d 16d 13d 27d 3d	10
**V14**	Hmawbi 5	2N6 – 2N6 – MSre – MS – Acclimation<br>34d 16d 13d 11d 3d	18
**V15**	Hmawbi Kauk Nyin Hmwe	N6D – 2N6 – MSre – MS –Acclimation<br>35d 13d 16d 14d 3d	82
**TK**	Tsukinohikari	N6D – 2N6 – MSre – MS – Acclimation<br>35d 13d 33d 21d 3d	31

The number of days (d) under each medium name indicates the duration of calli or regenerated plants kept on each medium. The number of regenerated plants for each variety were counted 3 days after acclimation. Calli of V5 and V7 did not produce any regenerated plants on MS medium.

A

Characteristics of Paw San Yin rice variety	
Origin	Myanmar
Life span	152-158 days
Plant height	160 cm
Ear bearing tillers	10-13 tillers
Length of panicle	9.5 cm
Grains per panicle	115
Filled grain percentage	85%
Grain measurement	Length 7.8 mm, Width 2.8 mm, Length/Width 2.78
1000 grain weight	28 g
Amylose	24.2
Grain appearance	Opaque
Eating quality	Good/Palatable
Yield	3550 kg/ha

B

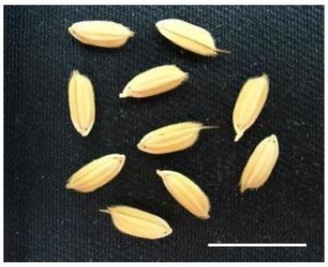

C

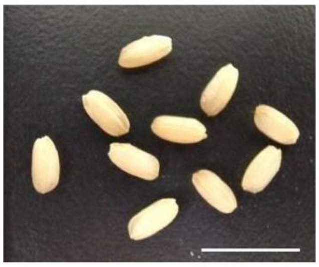

SUPPLEMENTARY FIGURE 1 | Characteristics of 'Paw San Yin' rice variety. **(A)** Characteristics of Paw San Yin rice variety. **(B)** Paw San Yin seed with husk. **(C)** Paw San Yin brown seed. Source of data of **(A)**: Myanma Agriculture Service (MAS), Ministry of Agriculture and Irrigation (MOAI), Myanmar. Scale bars in **(B)** and **(C)** = 1 cm.

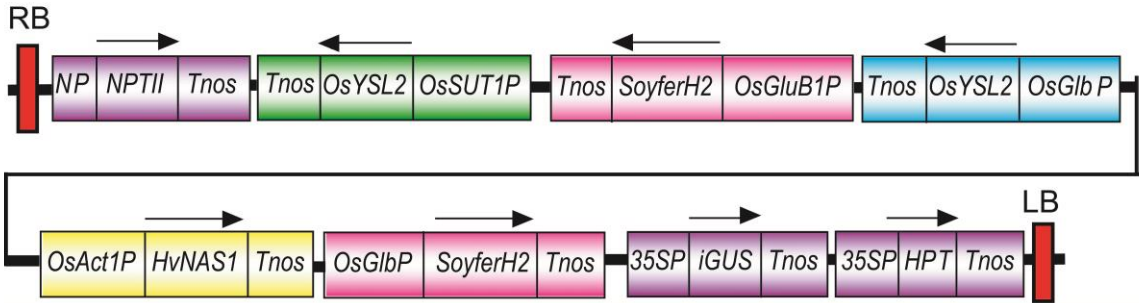

SUPPLEMENTARY FIGURE 2 | Fer-NAS-YSL2 gene cassette introduced into Paw San Yin rice variety.

Arrows show the direction of transcription. RB, right border; LB, left border; *NP*, *Agrobacterium tumefaciens* nopaline synthase gene (AF485783) promoter region; *NPTII*, neomycin phosphotransferase II gene (AF485783); *OsSUT1P*, promoter region of the rice sucrose transporter gene *OsSUT1* (D87819); *OsYSL2*, rice Fe (II)-NA and Mn (II)-NA transporter gene (AB126253); *OsGluB1P*, 2.3-kb promoter region of the *OsGluB1* gene (AY427569); *SoyferH2*, soybean *Ferritin* gene (AB062754); *OsAct1P*, promoter region of the rice *OsActin1* gene (Os03g0718100); *HvNAS1*, barley nicotianamine synthase 1 gene (AB010086); *OsGlbP*, promoter region of the 26 kDa *OsGlb1* gene (AY427575); *35SP*, cauliflower mosaic virus *35S* promoter (U28417); *iGUS*, *β*-glucuronidase gene with an intron (AF485783); *HPT*, hygromycin phosphotransferase gene (K01193). Each promoter and gene coding sequence set has *Tnos, A. tumefaciens* nopaline synthase gene terminator (AF485783), in the 3′-flanking region. pBIGRZ1 (Akiyama et al., 1997; Kawasaki, 2003) was used as vector back bone. This Fer-NAS-YSL2 vector was developed by Masuda et al. (2012).

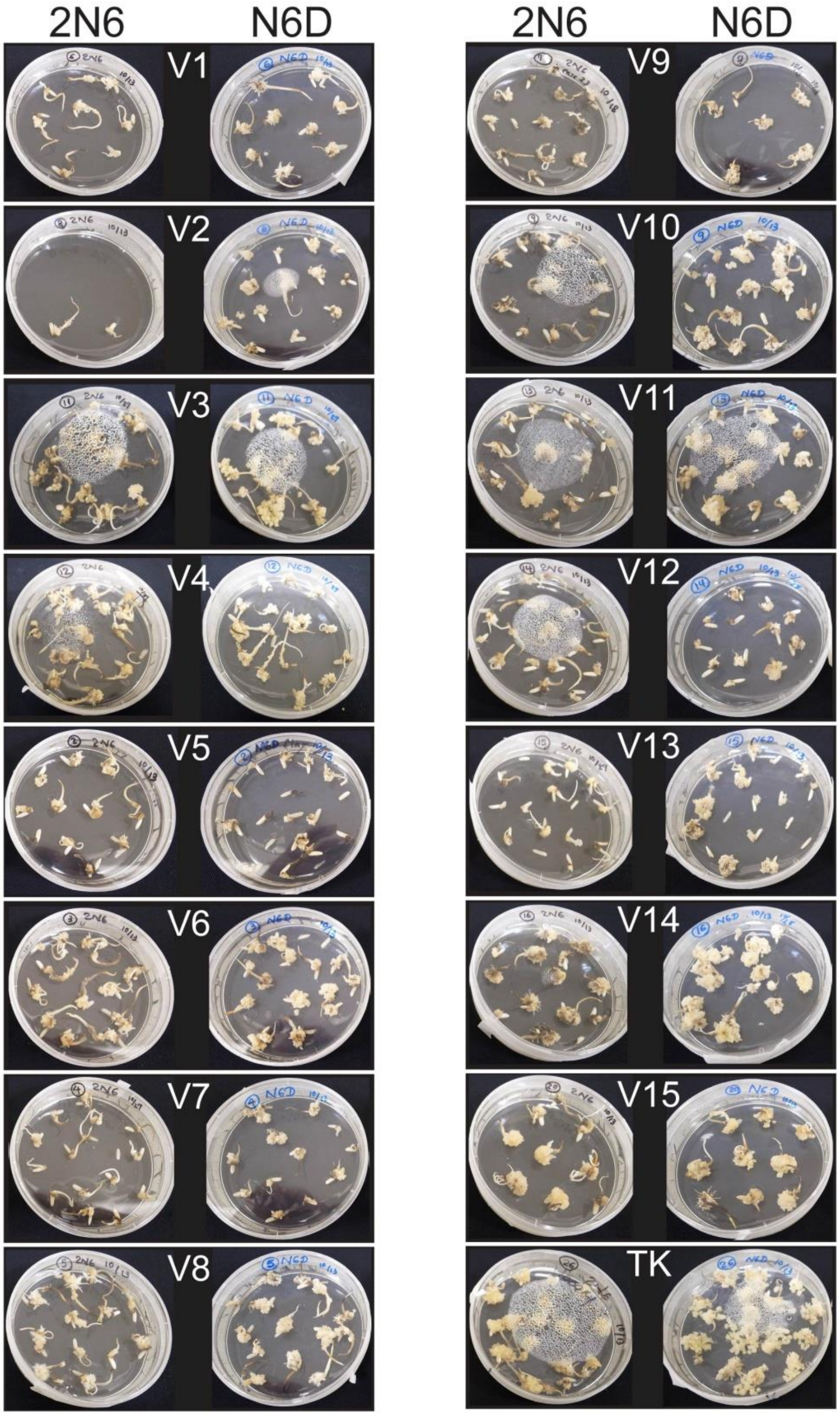

SUPPLEMENTARY FIGURE 3 | Callus induction tests on diverse Myanmar rice varieties and Tsukinohikari 27 days after germination (DAG) on N6D or 2N6 medium.

V1 to V15 shown on figure represent the variety numbers described in **Table 1**. TK, Tsukinohikari. Each variety was tested in both N6D (right) and 2N6 (left) media. Photographs were taken 27 DAG.

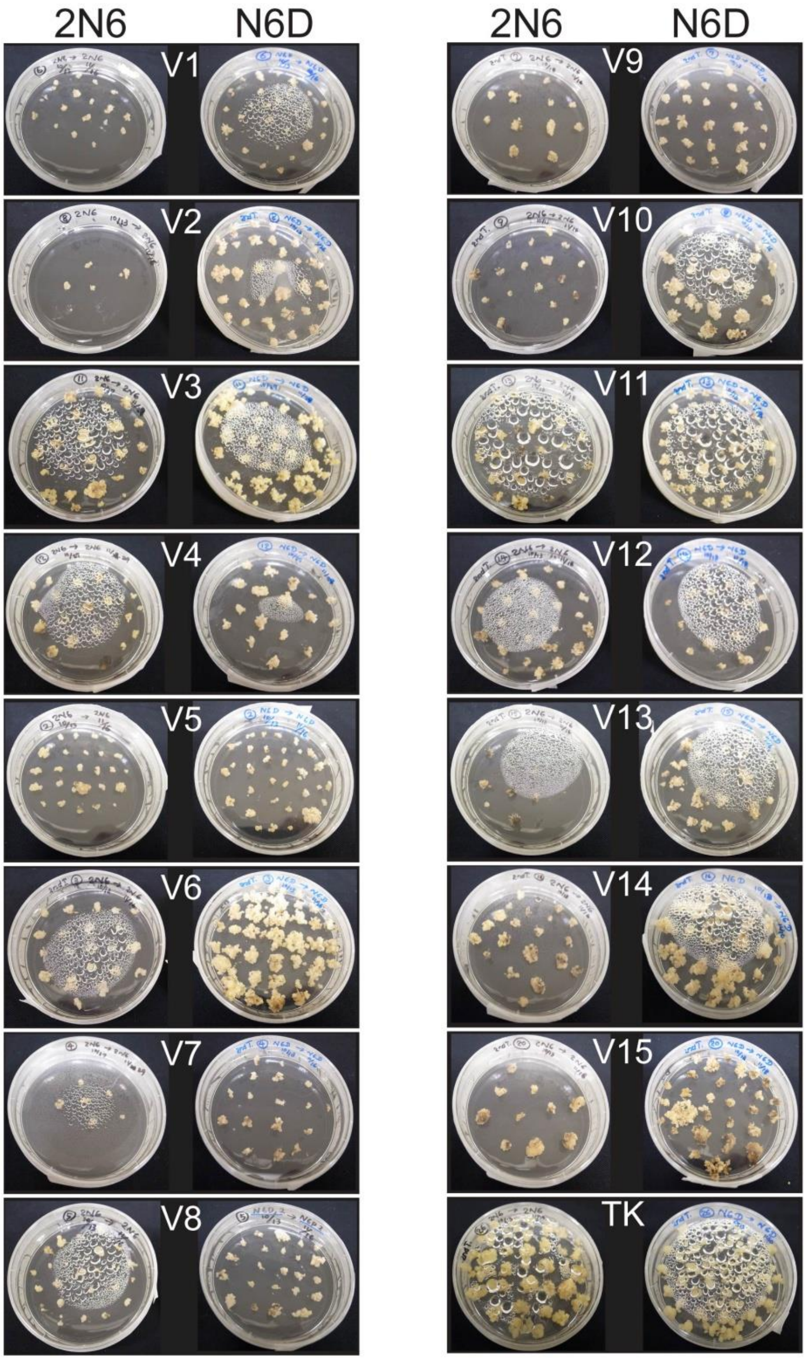

SUPPLEMENTARY FIGURE 4 | Callus growth tests on diverse Myanmar rice varieties and Tsukinohikari 19 days after transferring (DAT) on N6D or 2N6 medium.

V1 to V15 shown on figure represent the variety numbers described in **Table 1**. TK, Tsukinohikari. Each variety was tested on both N6D (right) and 2N6 (left) media. Photographs were taken 19 DAT from first N6D or 2N6 medium (Callus induction step) to second N6D or 2N6 medium (Callus growth step).

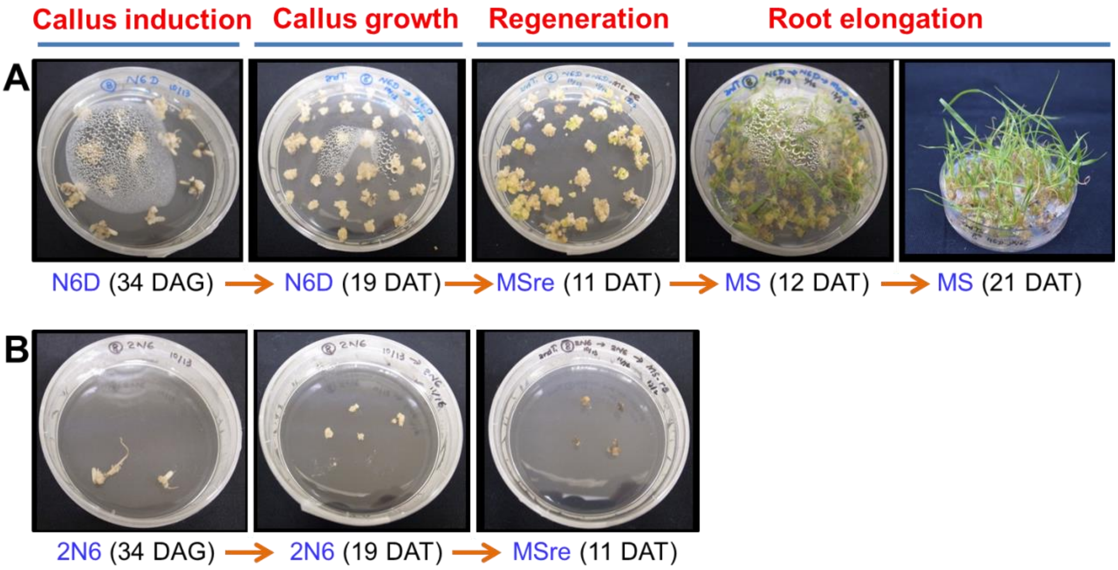

SUPPLEMENTARY FIGURE 5 | Analysis of callus induction and regeneration efficiency in Ayar Min (V2).

(A) Tissue culture in the N6D-N6D-MSre-MS medium combination. **(B)** Tissue culture in the 2N6-2N6-MSre medium combination. DAG, days after germination; DAT, days after transferring. The numerals shown inside parentheses mean DAG or DAT when photograph was taken on each medium.

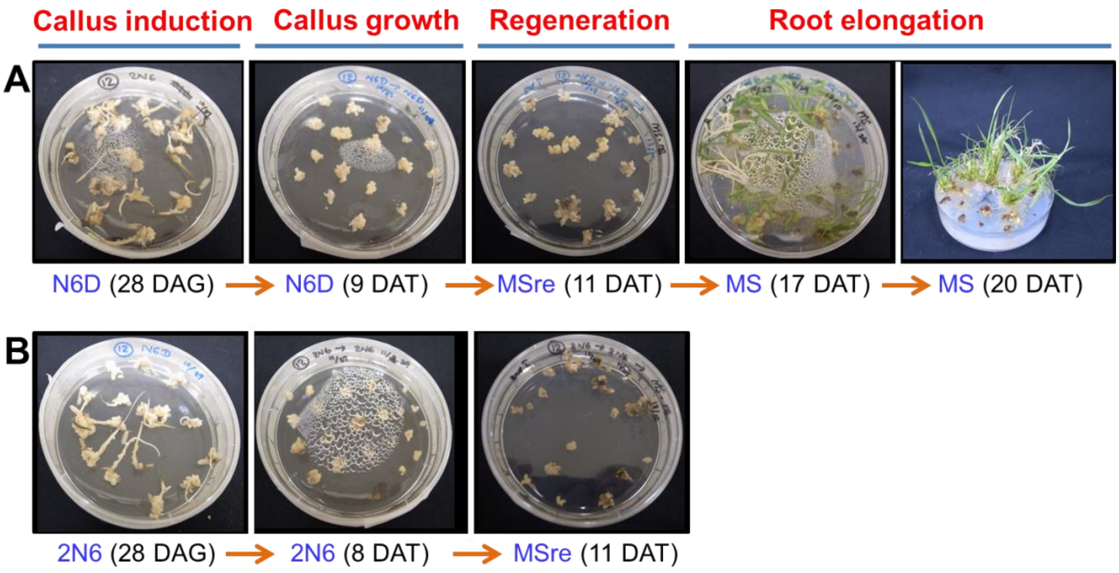

SUPPLEMENTARY FIGURE 6 | Analysis of callus induction and regeneration efficiency in Shwe War Htun (V4).

(A) Tissue culture in the N6D-N6D-MSre-MS medium combination. **(B)** Tissue culture in the 2N6-2N6-MSre medium combination. DAG, days after germination; DAT, days after transferring. The numerals shown inside parentheses mean DAG or DAT when photograph was taken on each medium.

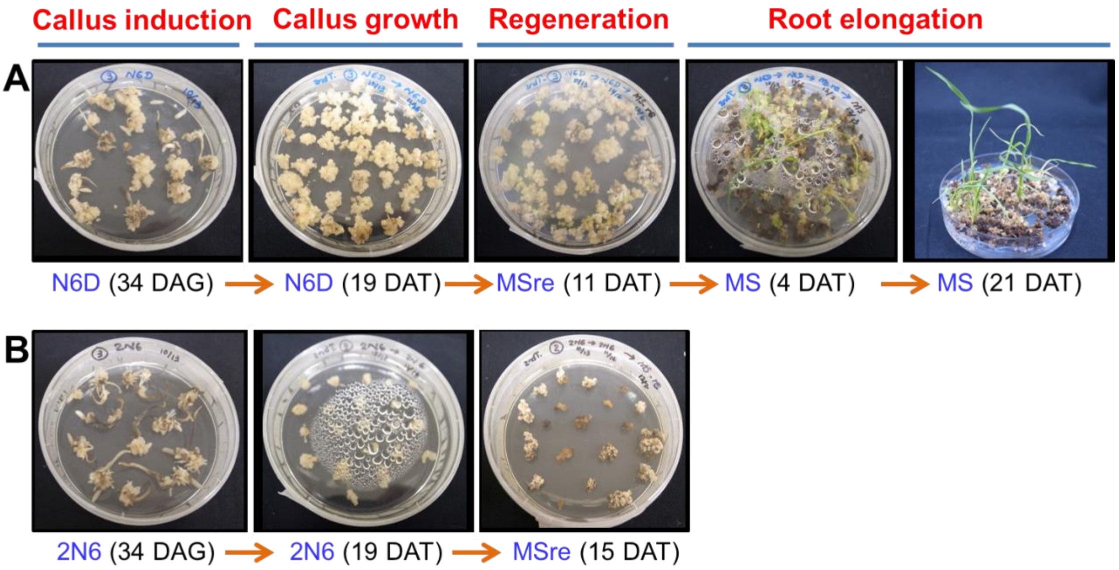

SUPPLEMENTARY FIGURE 7 | Analysis of callus induction and regeneration efficiency in Yezin Lone Thwe (V6).

(A) Tissue culture in the N6D-N6D-MSre-MS medium combination. **(B)** Tissue culture in the 2N6-2N6-MSre medium combination. DAG, days after germination; DAT, days after transferring. The numerals shown inside parentheses mean DAG or DAT when photograph was taken on each medium.

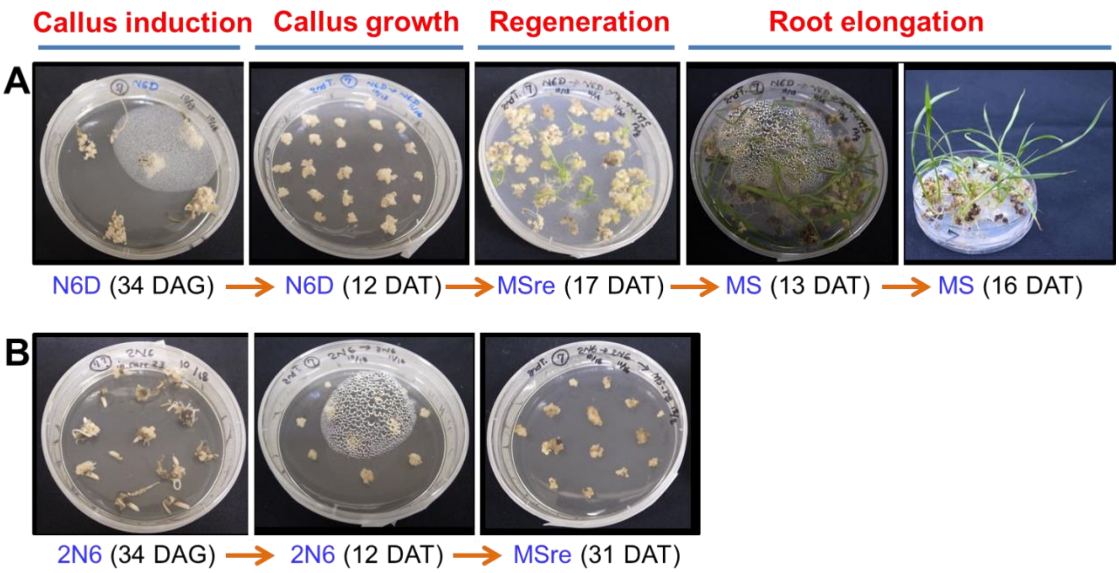

SUPPLEMENTARY FIGURE 8 | Analysis of callus induction and regeneration efficiency in Sin Nwe Yin (V9).

(A) Tissue culture in the N6D-N6D-MSre-MS medium combination. **(B)** Tissue culture in the 2N6-2N6-MSre medium combination. DAG, days after germination; DAT, days after transferring. The numerals shown inside parentheses mean DAG or DAT when photograph was taken on each medium.

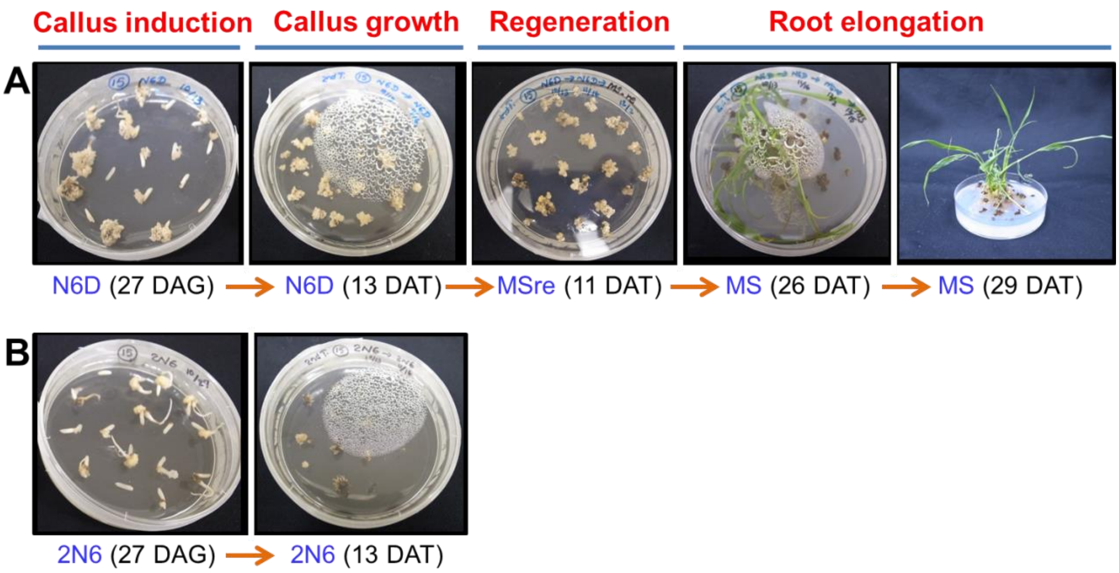

SUPPLEMENTARY FIGURE 9 | Analysis of callus induction and regeneration efficiency in Hmawbi 4 (V13).

(A) Tissue culture in the N6D-N6D-MSre-MS medium combination. **(B)** Tissue culture in the 2N6-2N6 medium combination. DAG, days after germination; DAT, days after transferring. The numerals shown inside parentheses mean DAG or DAT when photograph was taken on each medium.

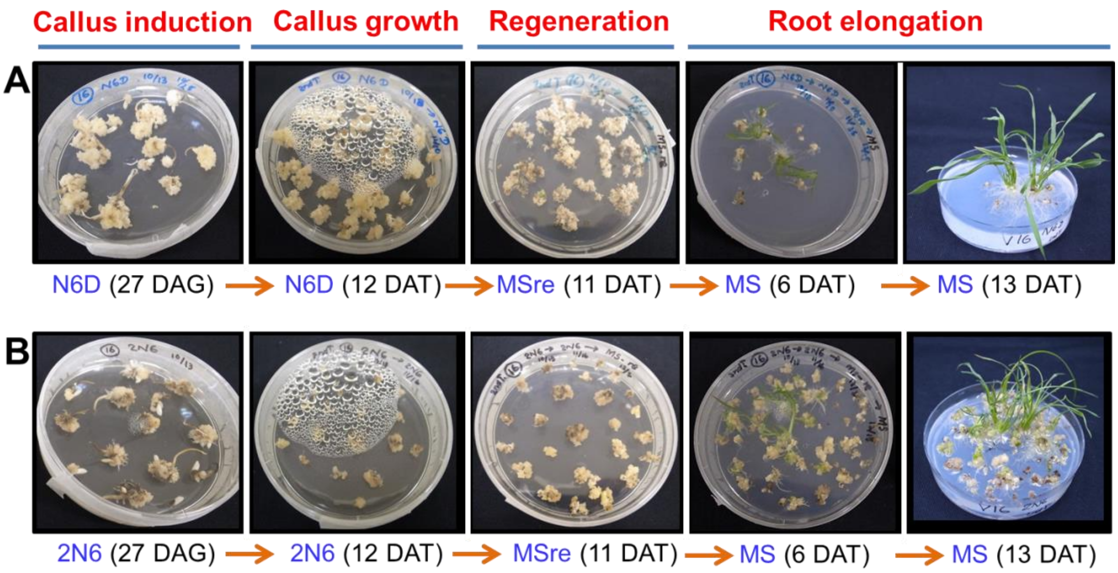

SUPPLEMENTARY FIGURE 10 | Analysis of callus induction and regeneration efficiency in Hmawbi 5 (V14).

(A) Tissue culture in the N6D-N6D-MSre-MS medium combination. **(B)** Tissue culture in the 2N6-2N6-MSre-MS medium combination. DAG, days after germination; DAT, days after transferring. The numerals shown inside parentheses mean DAG or DAT when photograph was taken on each medium.

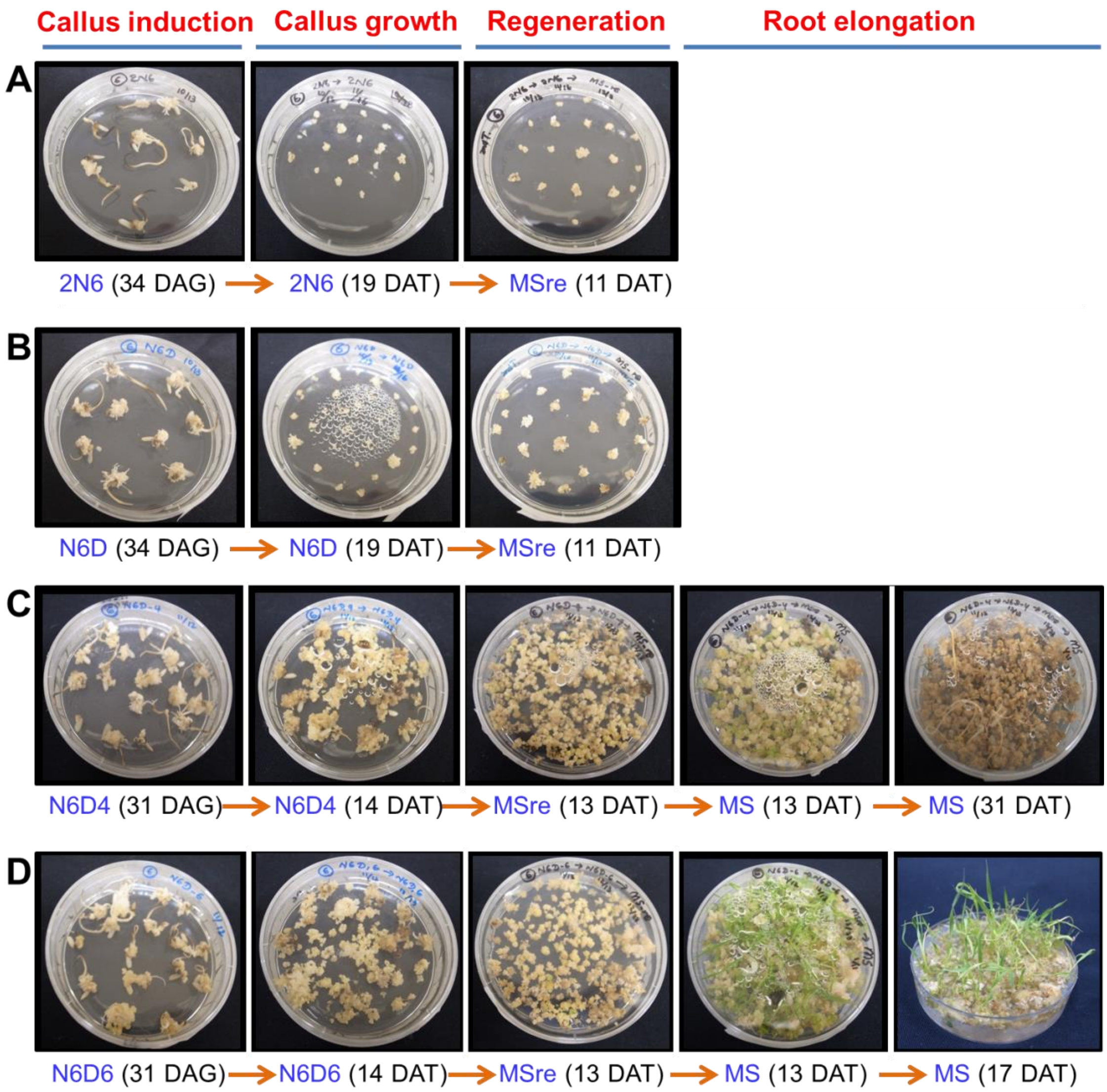

SUPPLEMENTARY FIGURE 11 | Analysis of callus induction and regeneration efficiency in Kyaw Zay Ya (V1).

(A) Tissue culture in the 2N6-2N6-MSre medium combination. **(B)** Tissue culture in the N6D-N6D-MSre medium combination. **(C)** Tissue culture in the N6D4-N6D4-MSre-MS medium combination. **(D)** Tissue culture in the N6D6-N6D6-MSre-MS medium combination. N6D4, N6D media with 4 mg l^{-1} of 2,4-D solution; N6D6, N6D media with 6 mg l^{-1} of 2,4-D solution. DAG, days after germination; DAT, days after transferring. The numerals shown inside parentheses mean DAG or DAT when photograph was taken on each medium.

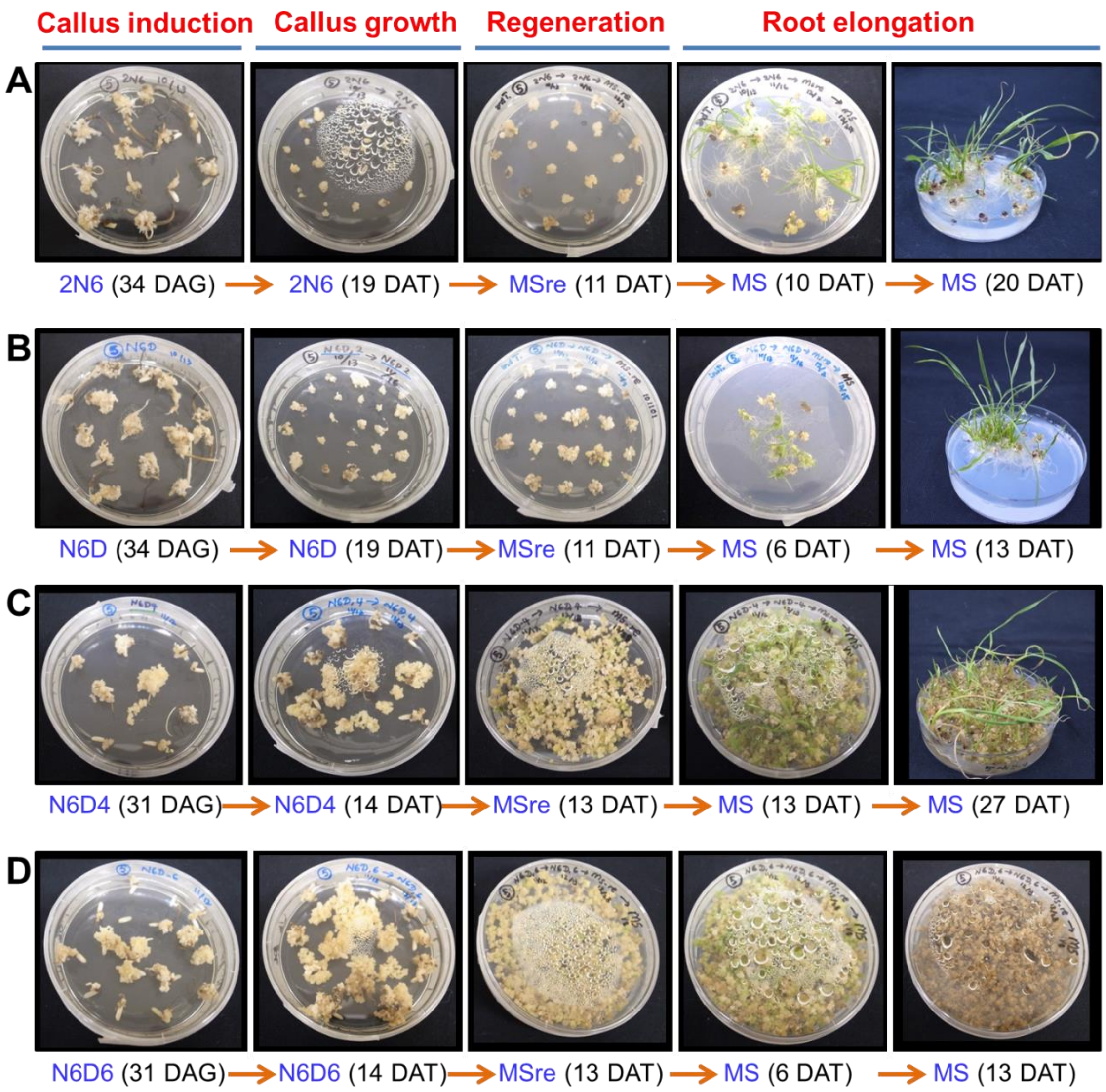

SUPPLEMENTARY FIGURE 12 | Analysis of callus induction and regeneration efficiency in Thee Htet Yin (V8).

(A) Tissue culture in the 2N6-2N6-MSre-MS medium combination. **(B)** Tissue culture in the N6D-N6D-MSre-MS medium combination. **(C)** Tissue culture in the N6D4-N6D4-MSre-MS medium combination. **(D)** Tissue culture in the N6D6-N6D6-MSre-MS medium combination. N6D4, N6D media with 4 mg l^{-1} of 2,4-D solution; N6D6, N6D media with 6 mg l^{-1} of 2,4-D solution. DAG, days after germination; DAT, days after transferring. The numerals shown inside parentheses mean DAG or DAT when photograph was taken on each medium.

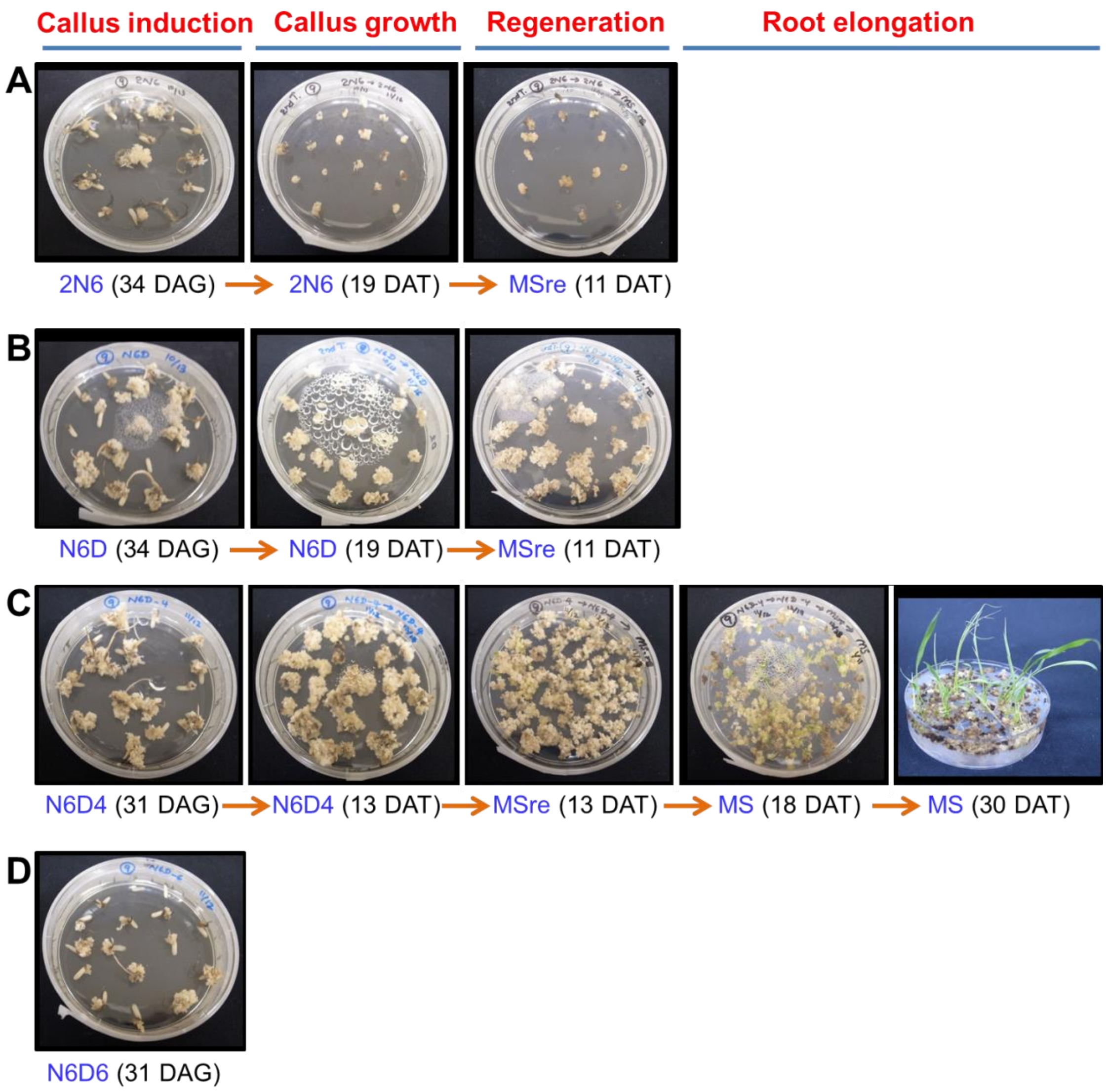

SUPPLEMENTARY FIGURE 13 | Analysis of callus induction and regeneration efficiency in Yadana Toe (V10).

(A) Tissue culture in the 2N6-2N6-MSre medium combination. **(B)** Tissue culture in the N6D-N6D-MSre medium combination. **(C)** Tissue culture in the N6D4-N6D4-MSre-MS medium combination. **(D)** Tissue culture in N6D6 medium. N6D4, N6D media with 4 mg l^{-1} of 2,4-D solution; N6D6, N6D media with 6 mg l^{-1} of 2,4-D solution. DAG, days after germination; DAT, days after transferring. The numerals shown inside parentheses mean DAG or DAT when photograph was taken on each medium.

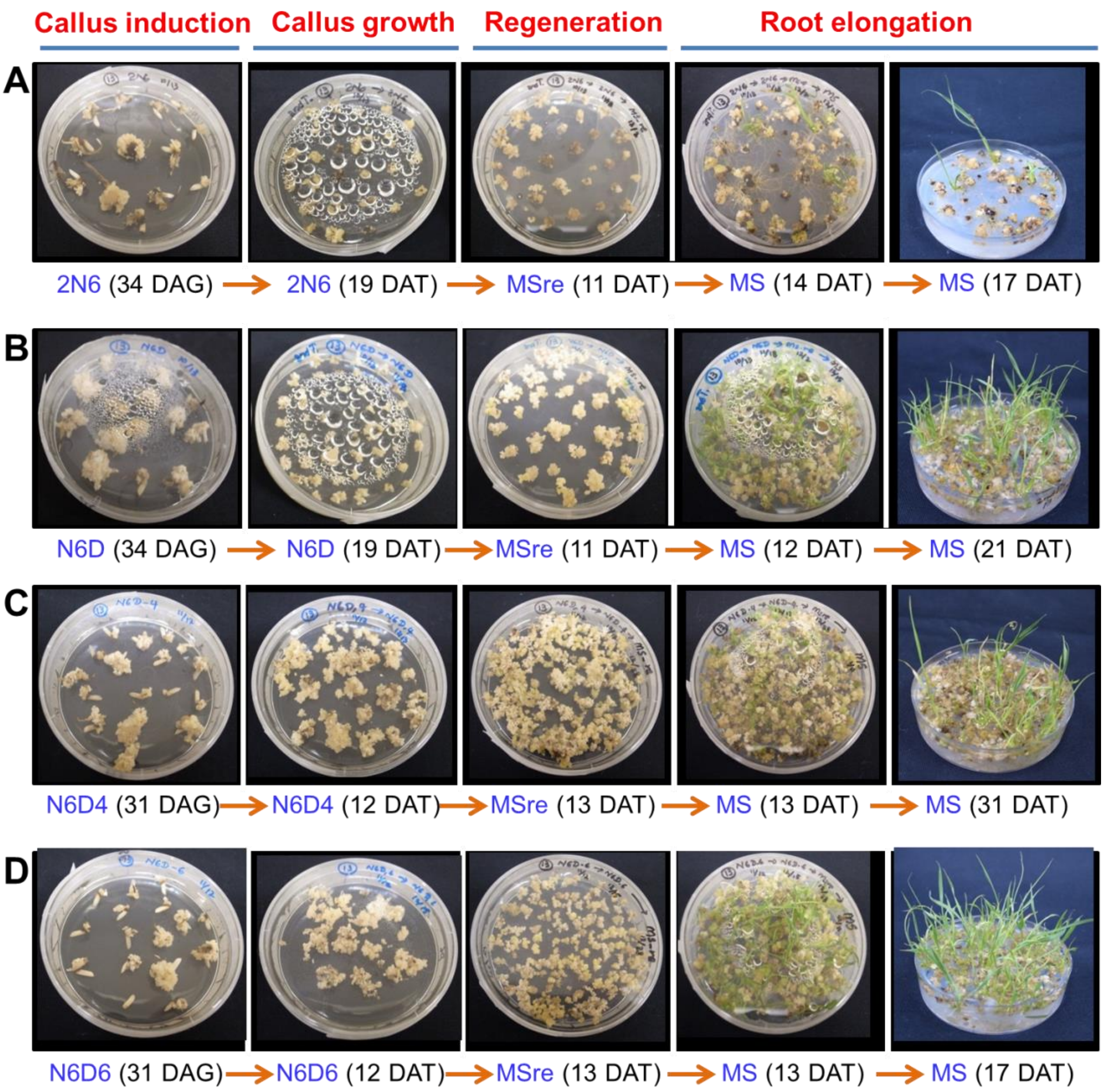

SUPPLEMENTARY FIGURE 14 | Analysis of callus induction and regeneration efficiency in Hmawbi 2 (V11).

(A) Tissue culture in the 2N6-2N6-MSre-MS medium combination. **(B)** Tissue culture in the N6D-N6D-MSre-MS medium combination. **(C)** Tissue culture in the N6D4-N6D4-MSre-MS medium combination. **(D)** Tissue culture in the N6D6-N6D6-MSre-MS medium combination. N6D4, N6D media with 4 mg l^{-1} of 2,4-D solution; N6D6, N6D media with 6 mg l^{-1} of 2,4-D solution. DAG, days after germination; DAT, days after transferring. The numerals shown inside parentheses mean DAG or DAT when photograph was taken on each medium.

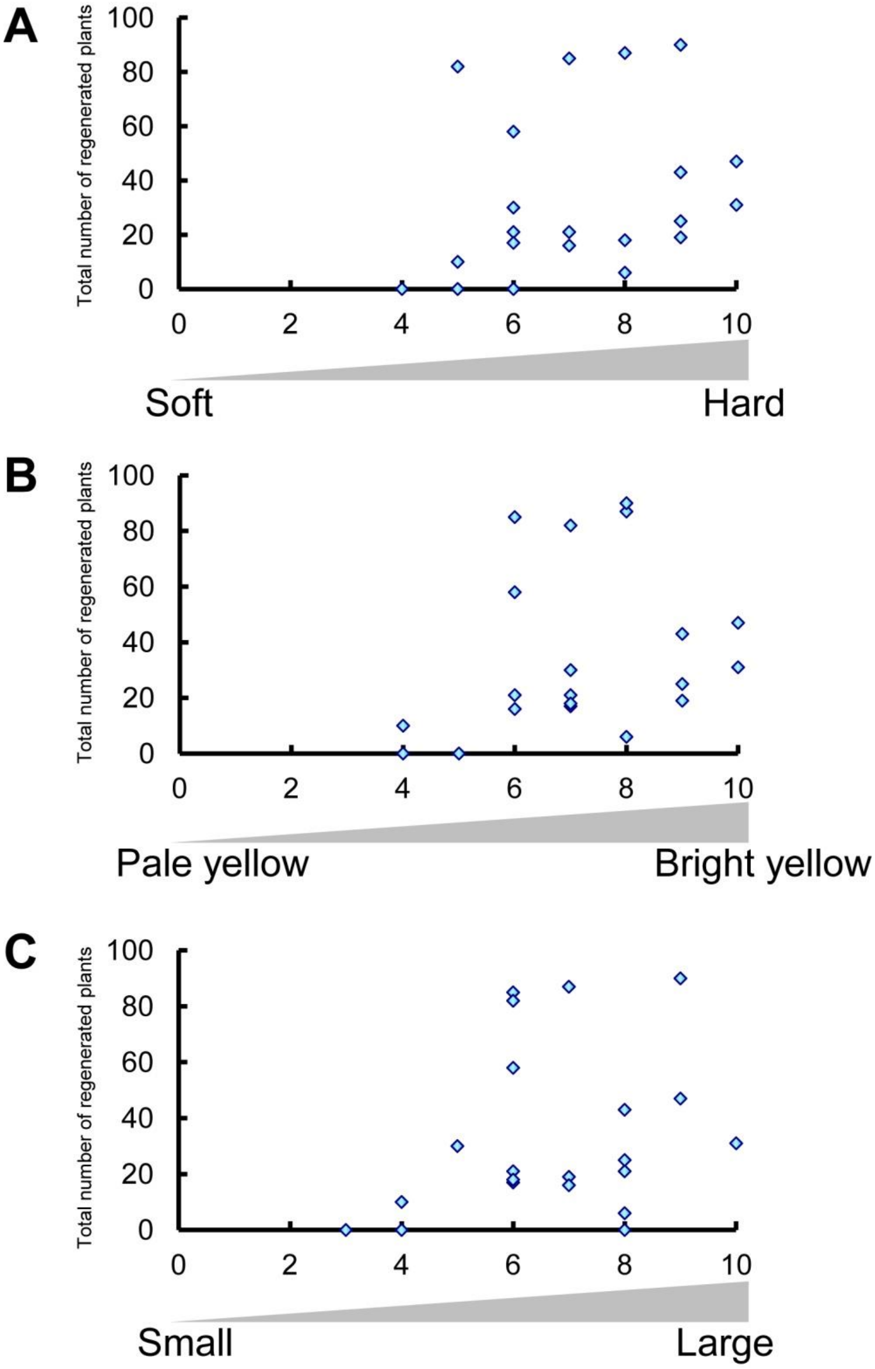

SUPPLEMENTARY FIGURE 15 | Correlation between callus condition and the regeneration rate.

Callus quality (hardness **(A)**, yellowness **(B)** and size **(C)**) were assessed. Calli were awarded scores of 1–10, which ranged from low to high quality based on callus quantity and quality.

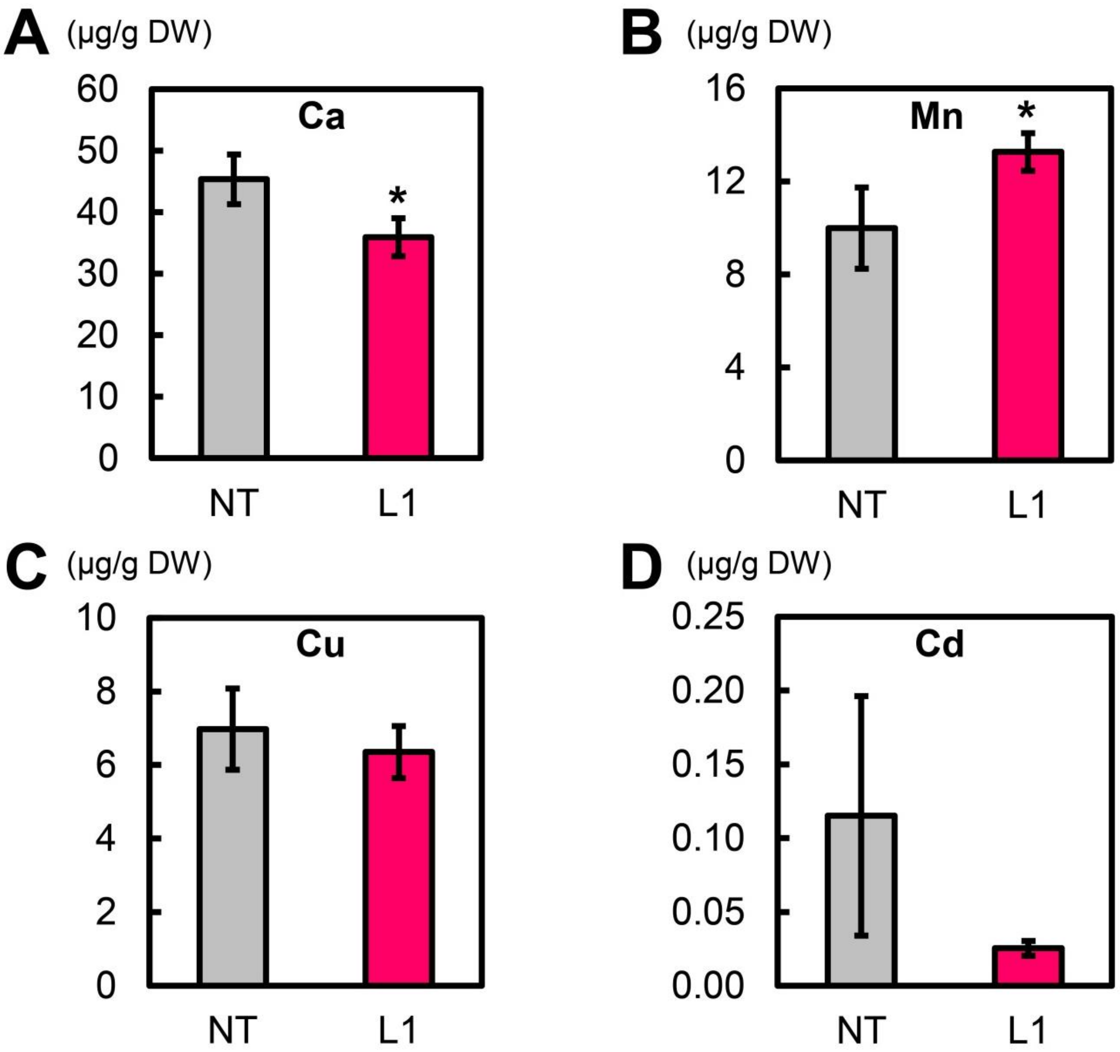

SUPPLEMENTARY FIGURE 16 | Metal concentrations in T_1 polished seeds of Paw San Yin-Fer-NAS-YSL2.

(A) Ca concentration. **(B)** Mn concentration. **(C)** Cu concentration. **(D)** Cd concentration. NT, non-transgenic Paw San Yin. L1, Paw San Yin-Fer-NAS-YSL2 transgenic line 1. Bars are means ± SE, $n = 3$. Asterisk (*) above the bars indicates significant differences between NT and L1 at $P < 0.05$, as determined by *t*-tests.

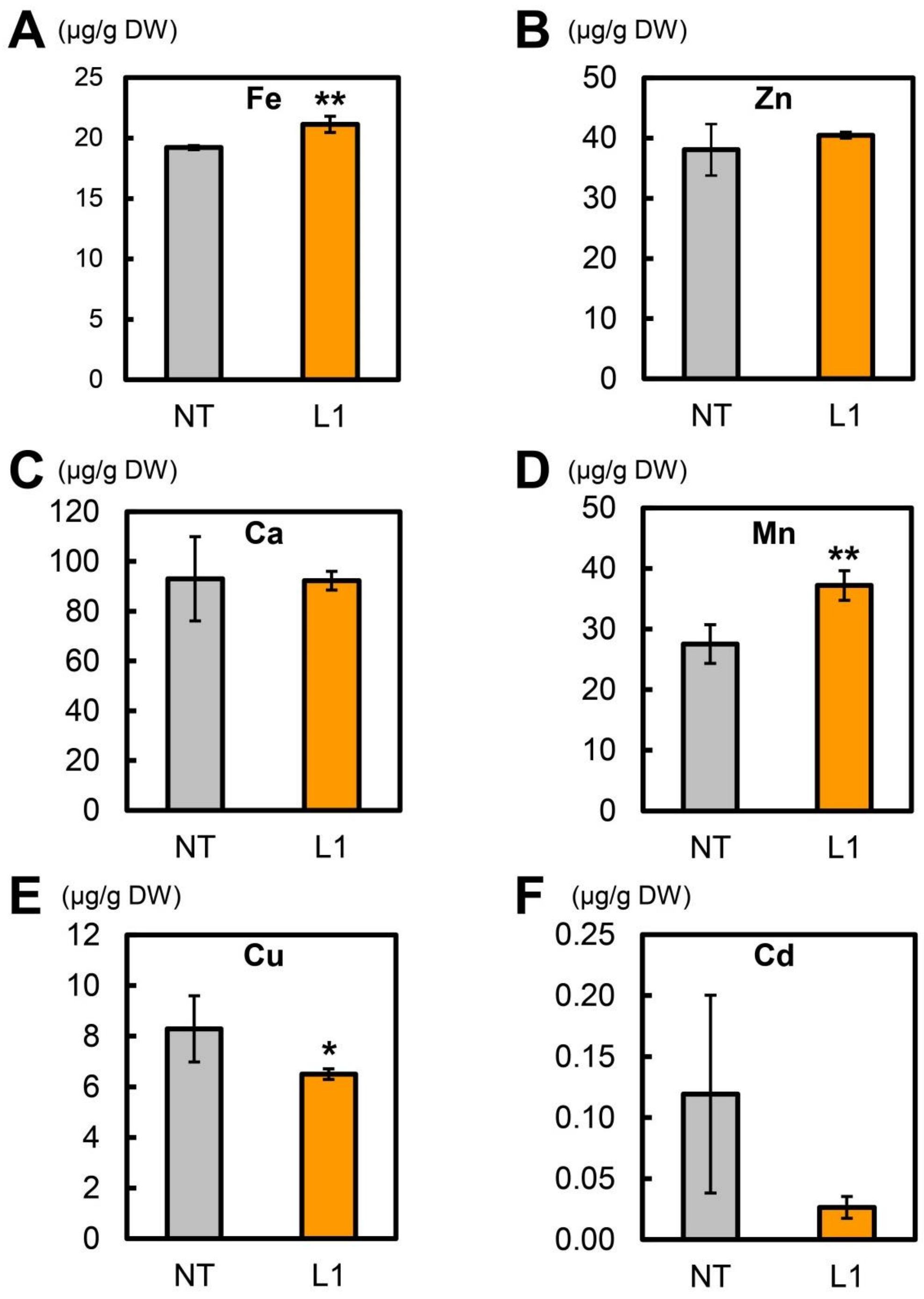

SUPPLEMENTARY FIGURE 17 | Metal concentrations in T_1 brown seeds of Paw San Yin-Fer-NAS-YSL2.

(A) Fe concentration. **(B)** Zn concentration. **(C)** Ca concentration. **(D)** Mn concentration. **(E)** Cu concentration. **(F)** Cd concentration. NT, non-transgenic Paw San Yin. L1, Paw San Yin-Fer-NAS-YSL2 transgenic line 1. Bars are means ± SE, $n = 3$. Asterisks (*) and (**) above the bars indicate significant differences between NT and L1 at $P < 0.05$ and $P < 0.01$, respectively, as determined by *t*-tests.

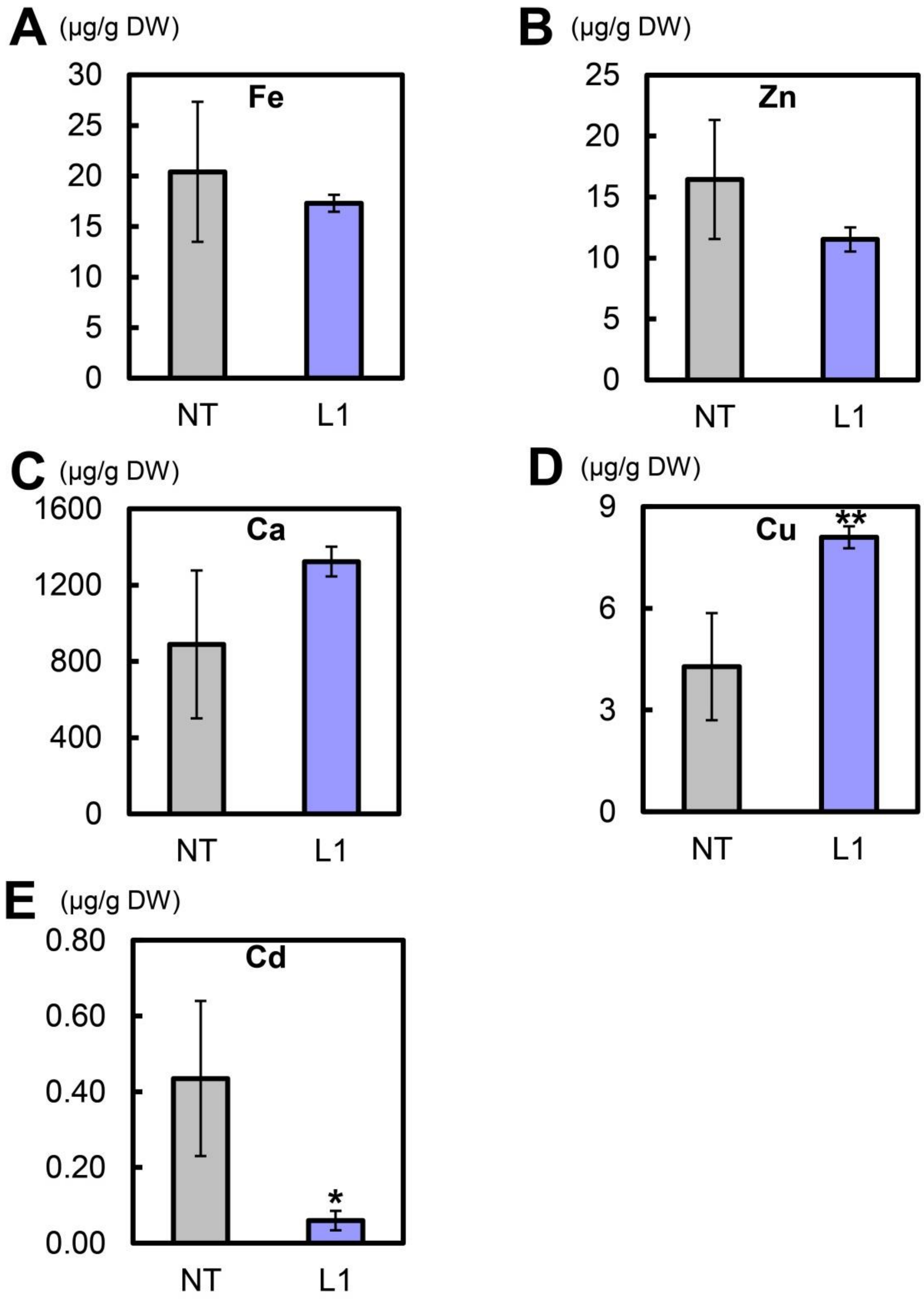

SUPPLEMENTARY FIGURE 18 | Metal concentrations in T_1 husk of Paw San Yin-Fer-NAS-YSL2.

(A) Fe concentration. **(B)** Zn concentration. **(C)** Ca concentration. **(D)** Cu concentration. **(E)** Cd concentration. Bars are means ± SE, $n = 3$. NT, non-transgenic Paw San Yin. L1, Paw San Yin-Fer-NAS-YSL2 transgenic line 1. Asterisks (*) and (**) above the bars indicate significant differences between NT and L1 at $P < 0.05$ and $P < 0.01$, respectively, as determined by *t*-tests.

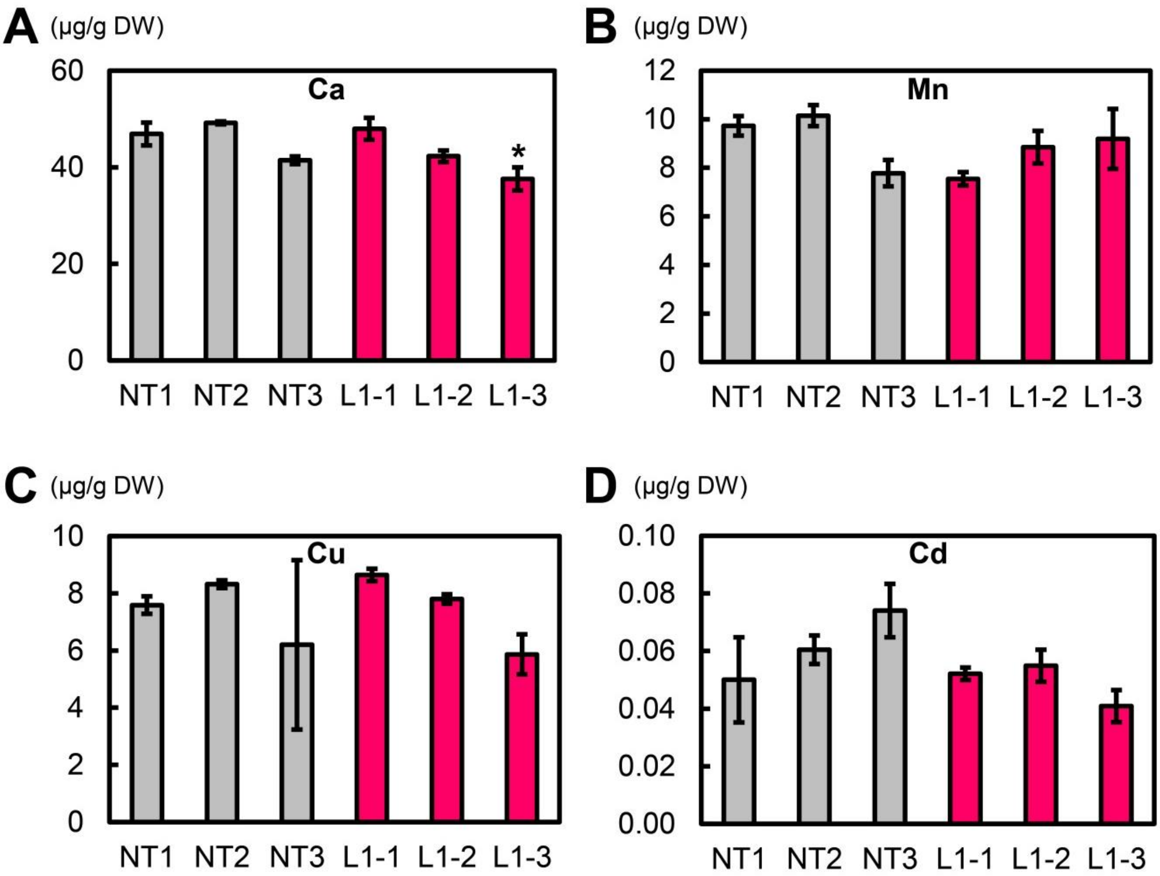

SUPPLEMENTARY FIGURE 19 | Metal concentrations in T_2 polished seeds of Paw San Yin-Fer-NAS-YSL2.

(A) Ca concentration. **(B)** Mn concentration. **(C)** Cu concentration. **(D)** Cd concentration. NT, non-transgenic Paw San Yin. L1-1, L1-2, and L1-3, Paw San Yin-Fer-NAS-YSL2 transgenic sublines. Bars are means ± SE, *n* = 3. Asterisk (*) above the bars indicates significant differences between NT and L1 sublines at $P < 0.05$, as determined by *t*-tests.

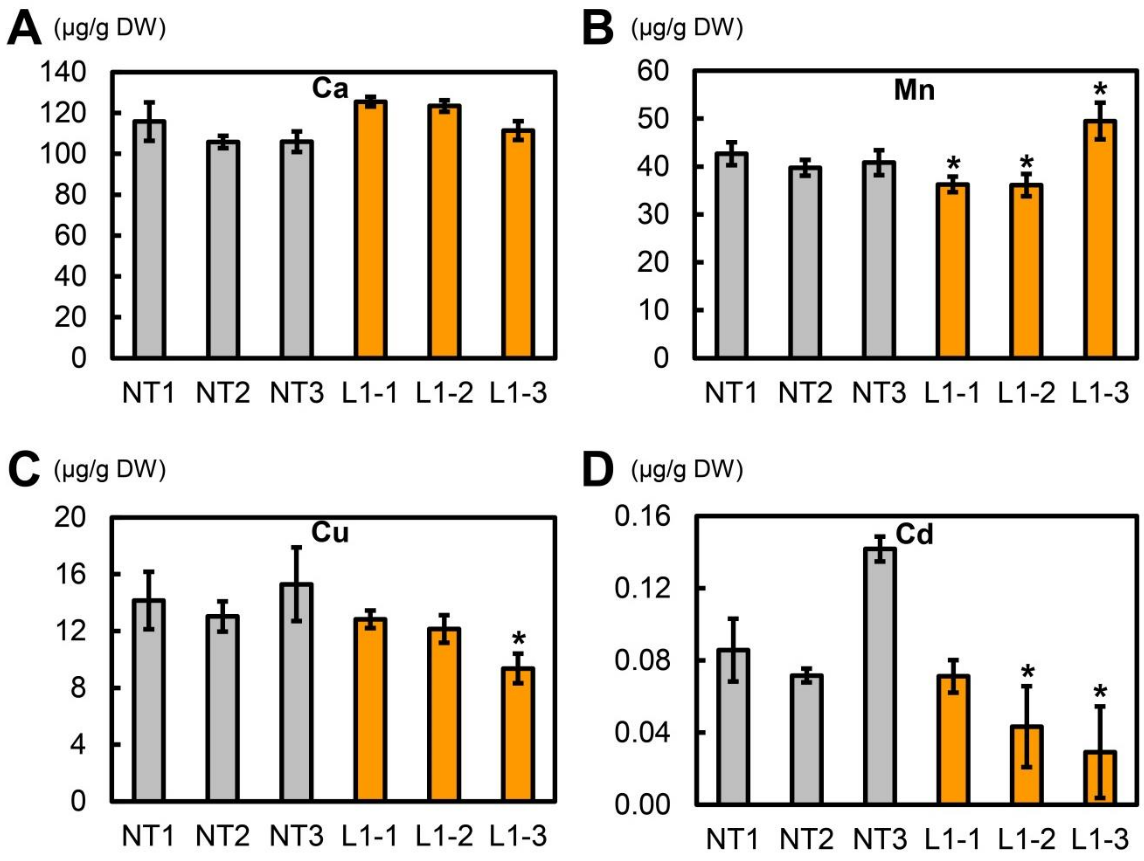

SUPPLEMENTARY FIGURE 20 | Metal concentrations in T_2 brown seeds of Paw San Yin-Fer-NAS-YSL2.

(A) Ca concentration. **(B)** Mn concentration. **(C)** Cu concentration. **(D)** Cd concentration. NT, non-transgenic Paw San Yin. L1-1, L1-2, and L1-3, Paw San Yin-Fer-NAS-YSL2 transgenic sublines. Bars are means ± SE, $n = 3$. Asterisk (*) above the bars indicates significant differences between NT and L1 sublines at $P < 0.05$, as determined by *t*-tests.

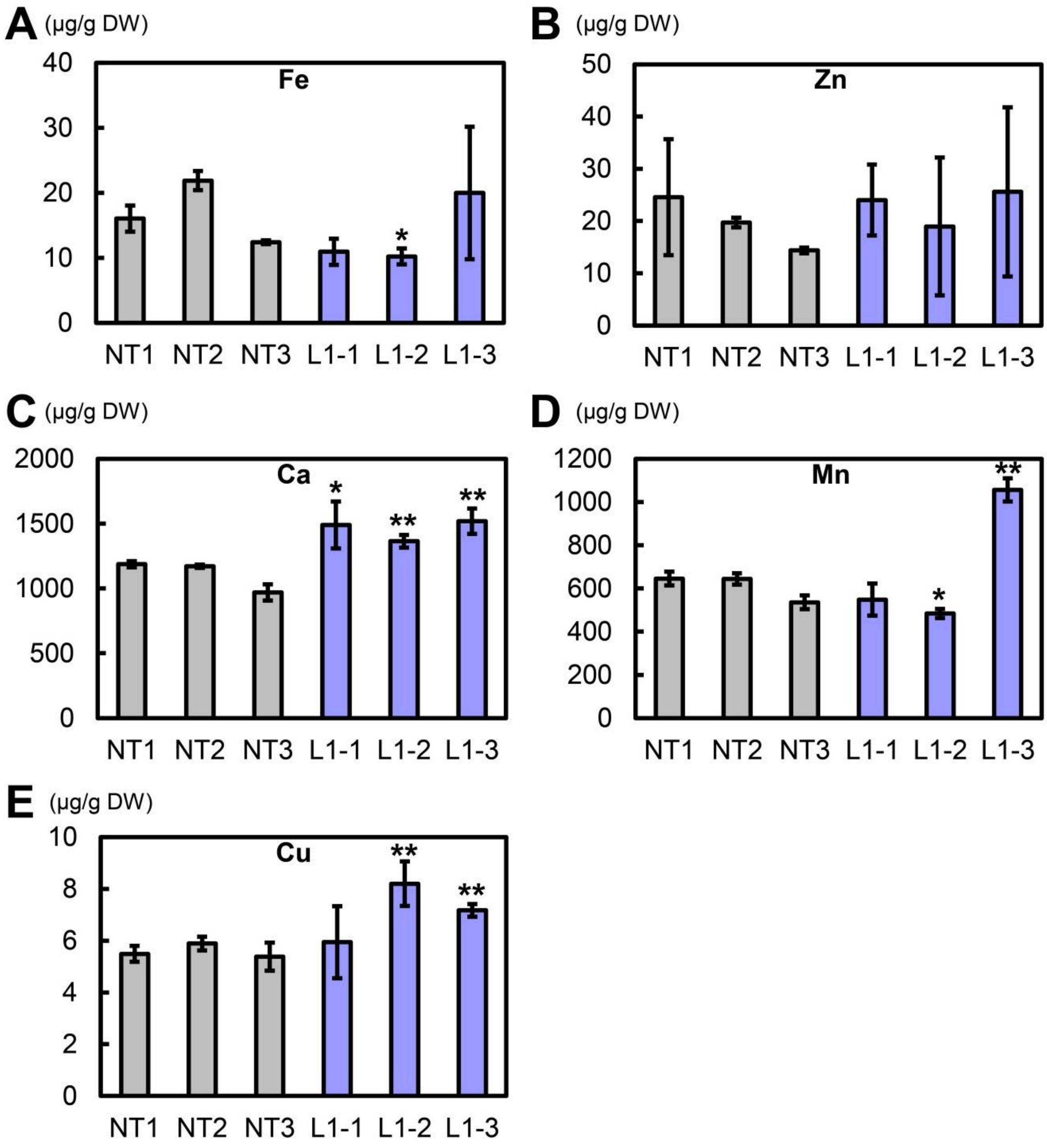

SUPPLEMENTARY FIGURE 21 | Metal concentrations in T_2 husks of Paw San Yin-Fer-NAS-YSL2.

(A) Fe concentration. **(B)** Zn concentration. **(C)** Ca concentration. **(D)** Mn concentration. **(E)** Cu concentration. NT, non-transgenic Paw San Yin. L1-1, L1-2, and L1-3, Paw San Yin-Fer-NAS-YSL2 transgenic sublines. Bars are means ± SE, $n = 3$. Asterisks (*) and (**) above the bars indicate significant differences between NT and L1 sublines at $P < 0.05$ and $P < 0.01$, respectively, as determined by *t*-tests.

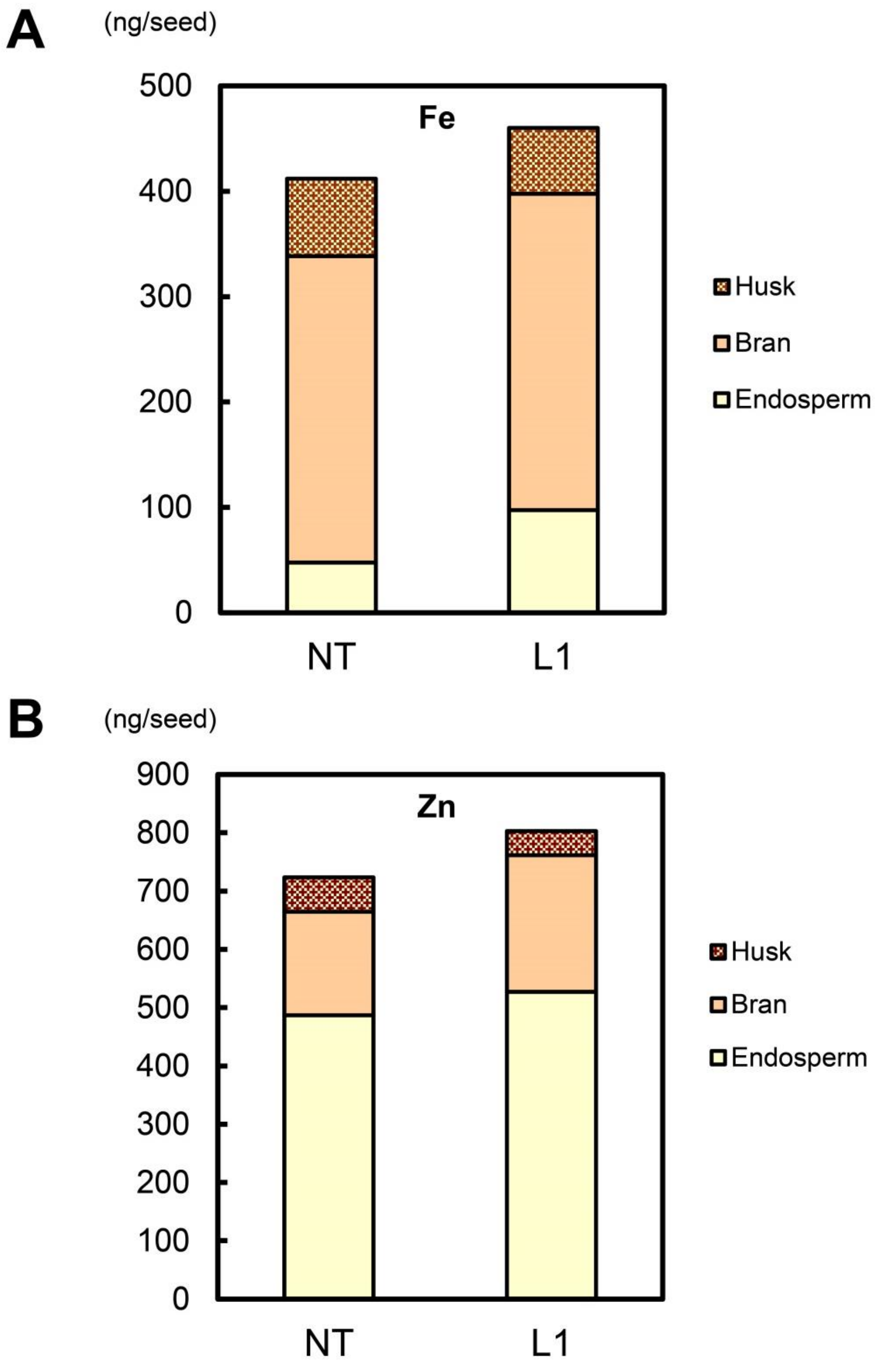

SUPPLEMENTARY FIGURE 22 | Fe and Zn contents per seed of Paw San Yin-Fer-NAS-YSL2 T_1 seed.

(A) Fe content in husk, bran, and endosperm per seed. **(B)** Zn content in husk, bran, and endosperm per seed. NT, non-transgenic Paw San Yin; L1, Paw San Yin-Fer-NAS-YSL2 transgenic line 1. Metal content of polished seed is shown as endosperm. Metal content of bran is calculated by subtracting metal content of polished seed from that of brown seed.
